# Extended Barrier Precautions vs Hand Hygiene Alone and Neonatal Sepsis in Intensive Care Patients: The BALTIC Cluster-Randomized Clinical Trial

**DOI:** 10.1001/jamanetworkopen.2026.12759

**Published:** 2026-05-15

**Authors:** Kirstin Faust, Franziska Strecker, Clara Haug, Ursula Felderhoff-Müser, Anja Stein, Reinhard Jensen, Mats Ingmar Fortmann, Janina Marißen, Christine Silwedel, Kirsten Brebach, David Frommhold, Christian Wieg, Georg Hillebrand, Barbara Naust, Esther Schmidt, Lutz Koch, Susanne Schmidtke, Arne Simon, Michael Zemlin, Sascha Meyer, Christopher Scholzen, Natascha Köstlin-Gille, Christian Gille, Ann-Carolin Longardt, Wolfgang Göpel, Manuel Krone, Stefanie Kampmeier, Dennis Nurjadi, Egbert Herting, Jan Rupp, Inke R. König, Christoph Härtel

**Affiliations:** 1 Department of Pediatrics, University Hospital Schleswig-Holstein, Lübeck, Germany; 2 German Center for Infection Research, Site Hamburg-Lübeck-Borstel-Riems, Lübeck, Germany; 3 Department of Pediatrics I, University of Duisburg-Essen, Essen, Germany; 4 Children’s Hospital Westküstenklinikum, Heide, Germany; 5 Department of Pediatrics, University Hospital Würzburg, Würzburg, Germany; 6 Children’s Hospital Memmingen, Memmingen, Germany; 7 Children’s Hospital Aschaffenburg-Alzenau, Aschaffenburg, Germany; 8 Children’s Hospital Itzehoe, Itzehoe, Germany; 9 Helios Children’s Hospital Schwerin, Schwerin, Germany; 10 Children’s Hospital Wilhelmstift Hamburg, Marienhospital Hamburg, Hamburg, Germany; 11 Neonatology, Asklepios Hospital Hamburg-Barmbek, Hamburg, Germany; 12 Department of Pediatric Hematology and Immunology, Saarland University Medical Center, Homburg, Germany; 13 Department for General Pediatrics and Neonatology, Saarland University Medical Center, Homburg, Germany; 14 SKK Klinikum, Franz-Lust Children’s Hospital, Karlsruhe, Germany; 15 Department of Neonatology, University Hospital Tübingen, Tübingen, Germany; 16 Department of Neonatology, University Hospital Heidelberg, Heidelberg, Germany; 17 Department of Pediatrics, University Hospital Schleswig-Holstein, Kiel, Germany; 18 Infection Control and Antimicrobial Stewardship Unit, University Hospital Würzburg, Würzburg, Germany; 19 Central Laboratory Unit, University Hospital Würzburg, Würzburg, Germany; 20 Institute of Medical Microbiology, University Hospital Schleswig-Holstein, Lübeck, Germany; 21 Infectious Disease Clinic, University Hospital Schleswig-Holstein, Campus Lübeck, Germany; 22 Institute of Medical Biometry and Statistics, University of Lübeck, Lübeck, Germany

## Abstract

**Importance:**

The effects of contact precautions (ie, gowns and gloves) for individual patients colonized with gram*-*negative (GN) drug-resistant bacteria on sepsis risk in neonates requiring intensive care remain to be clarified.

**Objective:**

To evaluate the noninferiority of standard hand hygiene disinfection vs standard hygiene disinfection plus extended barrier precautions for infants colonized with third-generation cephalosporins–resistant GN bacteria (3GCR-GNB).

**Design, Setting, and Participants:**

This cluster-randomized clinical trial was conducted from 2020 to 2023 in 12 German tertiary care neonatal intensive units caring for neonates with high risk for infections with GNB for 24 months, with crossover after 12 months. Follow-up and data curation were completed December 31, 2024, and statistical analysis was finalized on July 31, 2025.

**Intervention:**

The intervention was standard hand hygiene disinfection compared with current recommendations, ie, hygiene disinfection plus extended barrier precautions with gowns and gloves for routine care of infants colonized with 3GCR-GNB.

**Main Outcomes and Measures:**

The primary outcome was the rate of health care–associated GNB bloodstream infections (BSI) at infant level in all neonates requiring intensive care in the cluster, assuming 5% as noninferiority margin delta; secondary outcomes included transmission rates of 3GCR-GNB and rates of any infection.

**Results:**

The primary analysis was based on an overall sample size of 12 sites with crossover at 12 months, making 24 clusters with 9731 neonates. During the standard hand hygiene disinfection periods, 22 of 4699 infants (0.5%) developed GNB BSIs at infant level, compared with 25 of 5032 infants (0.5%) cared for during the extended barrier precaution periods (risk difference [RD], −0.03%; 95% CI, −0.43% to 0.38%; noninferiority *P* < .001). At least 1 nosocomial transmission with 3GCR-GNB was noted during 41 of 144 months in the intervention period and 54 of 144 months in the control period (RD, −9.03%; 95% CI, −27.79% to 9.74%), with involvement of 116 patients (2.5%) vs 149 patients (3.0%) (RD, −0.44%, 95% CI, −2.47% to 1.58%). The total rate of BSI was 2.1% in neonates during the intervention period vs 2.0% during the control period (RD, 0.12%; 95% CI, −1.39% to 1.64%).

**Conclusions and Relevance:**

In this cluster-randomized clinical trial, standard hand hygiene disinfection for the care of infants colonized with 3GC-GNB was noninferior to standard hygiene disinfection plus extended barrier precautions.

**Trial Registration:**

German Clinical Trials Register identifier: DRKS00019103

## Key Points

**Question:**

Is standard hand hygiene disinfection noninferior to extended barrier precautions (gloves and gown) for the routine care of neonates colonized with third-generation cephalosporins–resistant gram-negative bacteria in neonatal intensive care units?

**Findings:**

In this cluster-randomized crossover clinical trial including 12 sites and 9731 infants, standard hand hygiene was noninferior to extended barrier precautions with regard to infection rates with gram-negative bacteria and transmission events in neonatal units.

**Meaning:**

These findings support a shift toward more targeted, evidence-based practices for infection control, allowing resources to be focused where they are most effective.

## Teaser

This cluster-randomized clinical trial evaluates the noninferiority of standard hand hygiene disinfection vs standard hygiene disinfection plus extended barrier precautions for infants colonized with third generation cephalosporins–resistant gram-negative bacteria in neonatal intensive care units (NICUs).

## Introduction

Advances in perinatal medicine and neonatology have led to significantly improved survival rates for infants with very low birth weight (VLBW) and neonates who are critically ill and treated in neonatal intensive care units (NICUs). Endogenous factors, such as immaturity of skin and mucosal barriers and the need for intensive care measures (eg, vascular catheters, invasive ventilation), render these vulnerable infants at a high risk for health care–associated infections (eg, 15%-20% of bloodstream infections [BSI] in infants with VLBW are health care–associated).^1,2^ Health care–associated infections, particularly sepsis with gram-negative bacteria (GNB), are important causes of neonatal death and adverse long-term physical and neurodevelopmental outcomes.^3^ Therefore, there is a need to optimize and evaluate infection prevention and control (IPC) strategies targeting the prevention of nosocomial invasive infections and the spread of multidrug-resistant bacterial pathogens.

In this regard, extended barrier precautions (eg, contact precautions using patient-specific gowns and gloves) are commonly used IPC strategies. However, to our knowledge, the effectiveness of the combined use of gowns and nonsterile gloves has not been subject of a randomized clinical trial in infants colonized with multidrug-resistant organisms (MDROs) cared for in the NICU. The NICU typically includes preterm infants but also term infants with severe conditions (eg, congenital malformations, perinatal asphyxia). As a consequence of health care–associated infection outbreaks in German NICUs, the Commission for Hospital Hygiene and Infection Prevention affiliated at the Robert Koch Institute, Berlin (KRINKO) has recommended weekly colonization screening for all neonates requiring intensive care in NICUs since 2012. This screening aims to detect MDROs, such as acylureidopenicillins-resistant multidrug-resistant GNB and third-generation cephalosporin–resistant GNB (3GCR-GNB; equivalent to 2MRGN in the German nomenclature), GNB resistant to other classes of antibiotics (eg, fluorchinolones, carbapenems), methicillin-resistant *Staphylococcus aureus*, and bacteria with high epidemic potential.^4,5,6,7^ If screening results for 3GCR-GNB are positive, the KRINKO recommends adapted proactive hygiene interventions, ie, standard hand disinfection combined with extended barrier precautions using disposable, nonsterile gloves and long-sleeved gowns with wristbands during any patient contact.^5^ The protective clothing is intended to be used also in patients cared for in an incubator and needs to be changed between different patients. This strategy is time consuming for the attending health care workers. Time-trend analyses before and after the publication of recommendations in Germany did not demonstrate a change in health care–associated infection rates associated with screening relevant bacteria in infants with VLBW.^4,6^ The effectiveness of these additional barrier precautions remains unknown and builds the rationale for the multicenter Barrier Protection to Lower Transmission and Infection Rates With Gram-Negative Bacteria in Preterm Children (BALTIC) crossover cluster-randomized clinical trial.^5,6^ We hypothesize that standard hand hygiene disinfection (intervention) is noninferior to standard hand disinfection combined with gown and non-sterile glove use (control) for the routine care of neonates colonized with 3GCR-GNB. We selected the rate of health care–associated GNB BSIs in all neonates at the NICU level as primary end point, which is highly relevant for the affected infant^3^ and for the NICU as an ecosystem with different disease spectra and risk estimates. While single-center studies proposed that the use of nonsterile gloves may prevent health care–associated infections in vulnerable infants,^8,9,10^ adverse effects need to be considered (eg, reduced patient contact time and decreased quality of hand disinfection due to a false sense of security).^11,12^ The use of gowns and gloves increases costs and is a major source of health care–associated waste.^13,14^ From a sustainability perspective, the use of disposable gloves and gowns should be avoided wherever no clear clinical benefit has been demonstrated.^15^

## Methods

The BALTIC cluster-randomized clinical trial was approved by the institutional review board of the University of Lübeck and by the review boards of all participating sites. No identifying data about the infants were collected. Consent was not required because of this was a cluster-randomized trial with no randomization per patient. The trial protocol and statistical analysis plan are provided in Supplement 1. This study is reported following the Consolidated Standards of Reporting Trials (CONSORT) reporting guideline.

### Study Sites

The BALTIC study protocol was performed as outlined in the study protocol in Supplement 1^6^ and depicted in eFigure 1 in Supplement 2. In brief, 18 German NICUs with more than 200 admissions per year were screened for participation. All these sites had previously demonstrated their reliable performance in collaborative studies. Twelve sites (the clusters) were selected based on background information, which was collected through a structured interview, followed by a baseline observation, training and information sessions on standard hygiene precautions and proper gown and glove use, and a standardized audit performed by the clinical project management team either on-site or by remote meetings during the lockdowns due to the COVID-19 pandemic in 2020 to 2021. All neonates who required intensive care and regular colonization surveillance according to KRINKO were eligible as individual participants in the cluster. Clinician education and training was repeated before crossover after 12 months as controlled cointervention.^6^ To evaluate the adherence with IPC measures, direct observations were performed on a random basis by the project management team (audit at initiation of study and before crossover) and by local IPC nurses. Further mandatory in-service education sessions were performed on a regular basis by the local team. Audits, conducted as implemented in the study protocol, revealed high adherence to guidelines and comparable hand hygiene adherence during the study period. Management of transmissions and possible outbreaks were at the discretion of the local team based on NICU guidelines and therefore was different across the NICUs. The local standards regarding contact precautions in noncolonized infants and pandemic-related IPC policies were recorded at initiation of the study centers, after 12 months, and at the end of the study after 24 months.

### Randomization

We randomized the sequence of interventions for every site, ie, randomization of clusters stratified by site by a computerized simple sequence tool. The allocation to 2 trial group was performed by the study management and communicated to study sites, ie, to start with standard hand disinfection or to start with standard hand disinfection plus extended barrier precautions (gowns and gloves) for the care of infants colonized with 3GCR-GNB. The study groups were 12-month periods with crossover design, therefore 144 months of observation for each group.

### End Points

The primary end point was the rate of health care–associated GNB BSIs in all neonates who required intensive care and underwent regular colonization screening during the study period. Health care–associated GNB BSI was defined as infection occurring after 72 hours with at least 2 clinical signs and 1 elevated inflammatory marker, eg, increase in C-reactive protein more than 1 mg/dL (per local guideline; to convert to milligrams per liter, multiply by 10) and the detection of a GNB pathogen in the blood culture. The primary end point was assessed at infant level. Monthly measures were used to approximate infant-level confounders.

Key secondary end points were number of 3GCR-GNB transmission events, defined as colonization of 2 independent infants with the 3GCR-GNB species with identical resistogram patterns in a temporal (maximum 90 days) and spatial (potential contact in the intensive care unit) context; number of transmission events with any screening-relevant pathogen (as defined by the local study team); 3GCR-GNB BSI and total rate of BSI; rate of clinical sepsis (defined as 2 clinical signs of sepsis, 1 laboratory sign, no pathogen found in blood culture, and decision to treat for ≥5 days of antibiotics); number of initiated antibiotic cycles per month; hand disinfectant consumption; and yearly site-specific costs for gowns and gloves during study period. We also collected compulsory data on bed occupancy per NICU and staffing per shift during the study periods to account for organization and resource aspects.

### Quality Assurance and Data Management

During the study, quality assurance measures were implemented, including monitoring of site-specific data by the clinical project management before, during, and after the study to ensure that the study was conducted in accordance with the protocol, standard operating procedures, and the corresponding regulations of GCP, as supervised by study team members. In addition to the monitoring procedures, audits were carried out in accordance with the ICH-GCP Guidelines. The primary dataset was generated by the clinical project management of the study (K.F. and C. Härtel), entered into the central database, and curated after central monitoring.

### Sample Size Calculation

The statistical analysis plan is provided in Supplement 1. Sample size calculation and setting the noninferiority limit were based on assumptions of NICU rates of GNB BSI as the primary end point. We considered that the involved NICUs are highly variable in disease spectrum, number of infants with high-risk (eg, extremely preterm infants or term neonates requiring surgery), and in their potential for infant-to-infant bacterial transmission.^7^ We used previous data from BALTIC NICUs, in which GNB BSI rates ranged from 1.5% to 6.0% in infants with VLBW.^4,6^ Given the uncertainty on numbers of NICU-treated neonates at high susceptibility for GNB BSI apart from infants with VLBW, we estimated the mean GNB BSI rate to be 3% in the control clusters. With respect to the large heterogeneity across sites, we chose a noninferiority limit of 5%. Furthermore, the intracluster correlation was estimated to be 0.01 at maximum. We did not expect remarkable carryover effects, since medical caregivers were aware of the study period they were in and had received education sessions on hygiene measures before crossover. Data from 2 periods at 1 site were regarded as 2 independent groups. Striving for a power of 0.8 (1 − β) with a 1-sided significance level of α = .05, at least 18 patients per site and study group were required for analysis; thus, with 12 sites, a total of 432 patients were needed. To allow for a maximum dropout rate of 10%, at least 480 patients needed to be included. This sample size was to be achieved by collecting data for 12 months per period at every site. The trial was pragmatic with a preset timeframe of 24 months total per site and a crossover after 12 months within the same NICU.

### Statistical Analysis

The primary end point was analyzed using the intention-to-treat (ITT) principle, estimating a generalized estimating equations (GEE) model, assuming the independence correlation structure with robust sandwich estimators for the variance and taking correlation within clusters into account. Assuming a very low intracluster correlation, an independence working correlation structure was used. To obtain an estimate of the risk difference (RD), an identity link was used, and 1-sided 95% CIs are reported, given as the upper bound of a 90% CI. Sensitivity analyses included corresponding models adjusting for organizational aspects of the NICU, particularly shifts with understaffing, days of restricted bed occupancy, and additional workload in mixed NICUs by patients aged older than 28 days. Secondary end points were analyzed exploratorily with corresponding approaches using binomial and linear GEE models where appropriate. To simulate a per-protocol population, we excluded infants who were treated in clusters that were not fully adherent to randomization due to cocolonization of infants with 3GCR-GNB or with other bacteria or outbreak situations.

*P* values were 1-sided for the noninferiority test and 2-sided otherwise, and statistical significance was set at 5%. Data were analyzed using R version 4.5.1 (R Project for Statistical Computing) and SAS version 9.4 M8 (SAS Institute). Follow-up and data curation were completed December 31, 2024, and statistical analysis was finalized on July 31, 2025.

## Results

### Study Cohort

**Figure 1.**
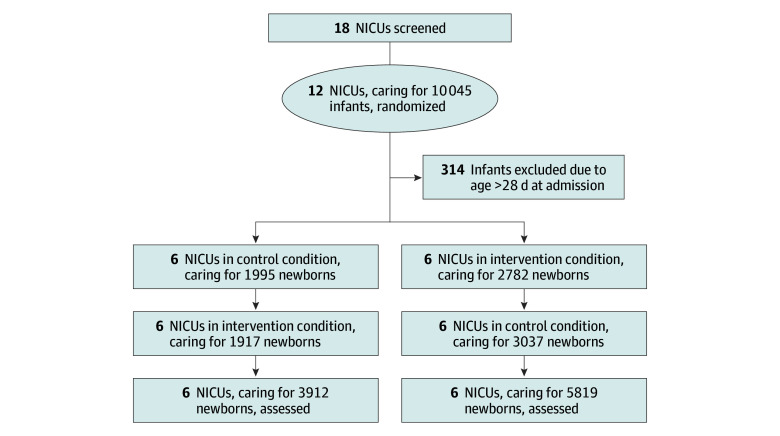
Flowchart of the BALTIC Study Enrollment and Randomization 3GCR-GNB indicates third-generation cephalosporin–resistant gram-negative bacteria; MDRO, multidrug-resistant organism; NICU, neonatal intensive care unit.

The primary analysis was based on an overall sample size of 12 sites, representing 24 clusters and 9731 neonates who were regularly screened for MDRO colonization. A total of 12 sites, including 10 045 infants, met all requirements for recruitment and randomization; 314 infants were excluded because they were older than 28 days at NICU admission (Figure 1). Eight NICUs were mixed wards with pediatric intensive care. The first center was randomized in October 2020, and the last center was randomized in June 2021. In 1 center, data collection was paused for 6 months due to a temporary intensification of hygiene measures during a nosocomial outbreak of *Acinetobacter baumannii*. Data collection and IPC policy according to randomization for 3GCR-GNB were resumed after the end of the outbreak. IPC policy in relation to randomization did not change during the COVID-19 lockdown; however, all centers implemented visitor restrictions and compulsory masking for parents and staff during this time.

**Figure 2.**
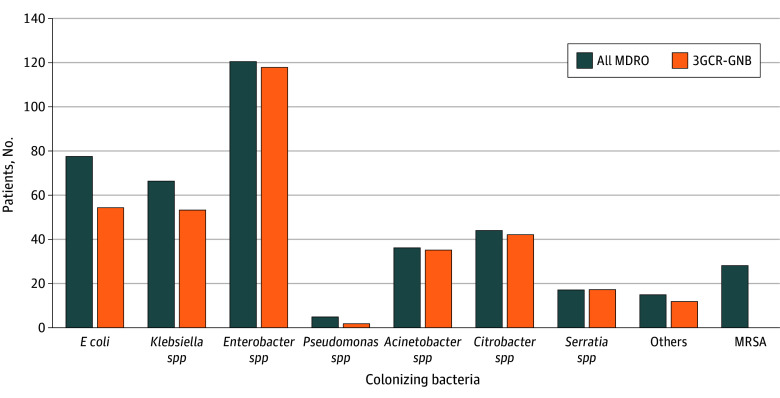
Bar Graph of the Number of Patients Colonized With Screening-Relevant Pathogens and Patterns of Antibiotic Resistance 3GCR-GNB indicates third-generation cephalosporin–resistant gram-negative bacteria; *E coli*, *Escherichia coli*; MRSA, methicillin-resistant *Staphylococcus aureus;* MDRO, multidrug-resistant organism.

A total of 4699 infants were cared in the 12 intervention clusters, and 5032 infants were cared for in the 12 control clusters *(*Table 1). Positive screening results for 3GCR-GNB were noted in 497 infants (5.1%), including 252 infants (5.5%) in the intervention periods and 245 infants (4.7%) in the control periods. Among these, full adherence to randomization was documented in 451 infants (90.8%), including 212 infants (84.3%) in the intervention months and 239 infants (97.7%) in the control months. Positive results in colonization screening were dominated by 3GCR-GNB, specifically *Enterobacter spp* (Figure 2).

**Table 1.** Characteristics of Clusters in Intervention and Control Groups

Measure	Standard hand hygiene disinfection	Extended barrier precautions	Overall
Clusters, No.	12	12	24
Individual participants in clusters with colonization screening, No.	4699	5032	9731
Colonized infants, No. (%)			
3GCR-GNB positive	252 (5.4)	245 (4.9)	497 (5.1)
Resistant GNB positive, any	329 (7.0)	290 (5.8)	619 (6.4)
Monthly infants in the NICU, median (IQR), No.	32 (24-62)	34 (25-68)	32 (25-65)
Days with blocked beds, median (IQR)	0 (0-23)	0 (0-7)	0 (0-13)
Shifts with documented understaffing according to guidelines, median (IQR), %^a^	4.4 (0-25.2)^b^	2.2 (0-16.1)^b^	3.6 (0-22.2)

^a^ Shift staffing according to the German guideline recommended by the Federal Joint Committee (Gemeinsamer Bundesausschuss) requires that infants with very low birth weight are cared for with 1 nurse per infant, 1 nurse for 2 infants, or 1 nurse for 4 infants based on their current clinical status and intensity level of care. Length of stay for cluster participants was similar during intervention compared with control (total hospital days: 61 805 vs 60 485 days).

^b^ This analysis was based on data of 9017 infants with missing values in the remainder population, 7680 infants were treated in clusters fully adherent to randomization. The nonadherence rate was significantly higher in the group with standard hand disinfection.

### Primary End Point

The incidence of GNB BSI was noninferior during the interventional period, with 22 events among 4663 infants (0.5%) compared with 25 events among 5032 infants (0.5%) during the control period (GEE; RD, −0.03%; 1-sided 95% CI, 0.38%; *P* < .001), per ITT analysis (Table 2). In the post hoc analyses of a simulated per-protocol population (9017 infants) the RD for GNB BSI rates was −0.1% (1-sided 95% CI, 0.17%) and −0.1% (1-sided 95% CI, 0.32%) when a potential correlation within clusters was additionally considered. When we excluded data from infants born in clusters with nonadherence, we confirmed noninferiority below our margin delta of 5% (RD −0.1%; 1-sided 95% CI, 0.17%).

**Table 2.** Study End Point Analysis

Measure	No. (%)			RD, % (1-sided 95% CI)
	Overall	Standard hand hygiene disinfection	Extended barrier precautions	
Patients, No.	9731	4699	5032	NA
Months, No.	288	144	144	NA
GNB BSI	47 (0.5)	22 (0.5)	25 (0.5)	−0.03 (0.38)
3GCR-GNB BSI	8 (0.1)	4 (0.1)	4 (0.1)	0.0 (0.13)
Any BSI	203	101 (2.1)	102 (2.0)	0.12 (1.64)
Clinical infection	557	292 (6.2)	265 (5.3)	0.88 (6.44)
Months with documented transmission of 3GCR-GNB	95 (33)	41 (28.5)	54 (37.5)	−9.03 (9.74)
Patients involved in transmissions with 3GCR-GNB	265 (2.7)	116 (2.5)	149 (3.0)	−0.44 (1.58)
Months with documented transmission of any MDRO	161 (55.9)	80 (55.6)	81 (56.3)	0.69 (23.45)
Patients involved in transmissions with any MDRO	816 (8.3)	424 (9.0)	392 (7.8)	1.26 (7.88)
Months with started reserve antibiotics	197 (68.4)	98 (68.1)	99 (68.8)	−1.66 (17.54)
Started antibiotic cycles, median (IQR), mo	10 (6 to 20)	10 (6 to 20)	10 (6 to 21)	−7.04 (44.97)
Days of antibiotic therapy per 100 d in hospital, median (IQR)	16 (9 to 27)	15 (9 to 27)	17 (9 to 27)	−11.22 (33.09)
Site-specific use of disinfection per year, median (IQR), L	191 (107 to 208)	191 (152 to 201)	191 (175 to 210)	NA

Abbreviations: 3GCR-GNB, third-generation cephalosporin–resistant gram-negative bacteria; BSI, bloodstream infection; MDRO, multidrug-resistant organism; NA, not applicable; RD, risk difference.

Assuming that the intervention effect was the same over all levels of confounders, we found no meaningful differences in GNB BSI rates between groups after adjustment for restricted bed occupancy (RD, −0.07%; 1-sided 95% CI, 0.48%), staffing (RD, −0.16%; 1-sided 95% CI, 0.27%), and number of admissions per day (RD, −0.02%; 1-sided 95% CI, 0.45%).

In an alternative model, we allowed the intervention effect to vary over levels of confounders and post hoc included respective interaction terms. The estimated RDs for confounder values were −0.17% for the first quartile (Q1), −0.17% for Q2, and −0.16% for Q3 for restricted bed occupancy and −0.21% for Q1, −0.18% for Q2, and −0.07% for Q3 for staffing. Adjustment for the number of admissions per day revealed RDs of −0.01% at median and −0.13% at 95% percentile of admission.

### Secondary End Points

One outbreak involving at least 2 patients, including 1 BSI with the same 3GCR-GNB species, occurred in each group. Likewise, BSI events with 3GCR-GNB were identical, affecting 4 infants per group. At least 1 possible nosocomial transmission event with 3GCR-GNB was noted during 41 of 144 months of standard hand disinfection and 54 of 144 months of extended barrier precaution period (RD, −9.03%; 1-sided 95% CI, 9.74%) involving 116 patients (2.5%) vs 149 patients (3.0%) (RD, −0.44%, 1-sided 95% CI, 1.58%). We found no differences in any MDRO transmission or in rates of any BSI, clinical sepsis, or number of antibiotic cycles (Table 2). We noted a large center-to-center variability in the numbers of months with transmissions of any MDRO, with any transmission during study condition periods, transmissions with 3GCR-GNB, and incidences of any GNB BSI, any BSI, and clinical sepsis (eFigure 2 in Supplement 2). In line with this, center-specific antibiotic treatment rates of infants requiring intensive care ranged from 10 to 48 days per 100 days in hospital (eFigure 2 in Supplement 2). There was higher site-specific yearly costs for disposable gloves and gowns in the extended barrier group compared with the handwashing group (median [IQR] cost, €34 600 [€23 000-€75 500] vs €25 700 [€22 100-€59 700]). The pathogenic spectrum identified by colonization screening differed remarkably across sites, suggesting site-specific endemic flora (eFigure 3 in Supplement 2).

## Discussion

The BALTIC trial is the first controlled, multicenter, cluster-randomized clinical trial to our knowledge to evaluate the effectiveness of additional hygiene precautions using gloves and gowns in infants colonized with 3GCR-GNB and treated in NICUs. Regarding the rate of BSIs with GNB, standard hand disinfection (intervention) was noninferior to standard hand disinfection with extended barrier precautions using patient-specific gowns and gloves (control). In line with that, we found no significant differences in rates for either any BSI or clinical infection nor for transmission events of MDROs. The use of gowns and gloves is a major contributor to environmental burden related to intensive care^14^ and a significant driver of costs. Given the urgent need to develop green hospital strategies,^13,16^ the use of gloves and gowns should be discouraged when clinical evidence is lacking.

Newborn infants requiring intensive care may become colonized with MDROs.^5,6^ The main risks for colonization of MDROs in infants are system-level aspects at the hospital site and heterogeneity in NICU endemic flora.^7^ In the BALTIC trial, we confirmed large variability in site-specific antibiotic use, concentration of vulnerable infants, and staffing. Furthermore, a large proportion of infants in the NICU are born preterm and serve as reservoirs for nosocomial transmission, which is further facilitated by close proximity of infants or restricted bed capacity in many units.^17,18,19^ In line with this, the BALTIC trial and others^8,9,10^ suggest that gastrointestinal colonization and prevention of GNB infections (MDROs and non-MDROs) are due to other factors apart from barrier precautions, eg, the complexity of successfully integrating potential pathogens into the neonatal microbiome,^7^ that have not yet been uncovered. Future studies need to explore new and alternate colonization and transmission mechanisms.

Current evidence clearly underlines that standard rigorous hand disinfection with alcohol rubs is the cornerstone for preventing MDRO colonization in the NICU.^9,20^ Regular educational sessions and monitoring the adherence to hand hygiene guidelines are essential means for infection control.^21^ Active surveillance programs for MDROs and the targeted and temporary use of additional barrier precautions lead to the containment of MDRO transmission during outbreaks.^22^ Beyond active surveillance, NICUs have implemented regular environmental interventions, such as dedicated equipment, cleaning and disinfection, contact precautions, and fixed number of required staff, within their IPC policies. The combined use of these interventions may be effective for outbreak containment; however, studies in complex IPC bundles on NICUs make it difficult to determine the effect of any single measure.

According to German legislation, the KRINKO recommendations define the standard of care in IPC and therefore are implemented in most German NICUs.^23^ The BALTIC trial used a novel approach, as all 12 participating sites had a standardized active surveillance of MDRO, required NICU staffing ratios (per regulation of the German authorities), and continuous IPC education as controlled cointerventions. Therefore, we were able to isolate the effect of additional glove and gown use in infants colonized with 3GCR-GNB on total infection risk at the NICU level on a cluster level. The 3GCR-GNB colonization in the BALTIC study population (6.3%) was lower than the 10% expected based on previous publications.^7^ Possible explanations include the increased awareness due to participation in a study (ie, Hawthorne effect^24^) and different study populations with reduced length of stay as compared with infants with VLBW.

The use of gloves may reduce hand hygiene adherence due to false beliefs of security, as pathogens easily contaminate, and gloves cannot be disinfected between manipulations of a preterm infant in an incubator.^25^ In addition, extended hygiene measures may reduce patient contact time and interfere with other priorities of individualized developmental care. Beyond the important need to reduce hospital waste, financial costs are critical, and we found that costs were one-third higher for disposable protective equipment during extended barrier precaution period than during the period with standard hand disinfection alone. We estimate that by forgoing disposable gloves and gowns for handling preterm infants colonized with 3GCR-GNB in German NICUs, €4 million could be saved annually. In line with the available evidence, we conclude from the BALTIC trial that the routine use of gowns and gloves in infants colonized with 3GCR-GNB is not necessary outside outbreak situations.

The BALTIC study has some strengths. The multicenter design with crossover and standardized trainings represent a practical setting; therefore, the data are generalizable for the context of German NICUs. We took correlations within clusters into account and addressed confounding variables for hygiene adherence, such as staffing. The center-to-center variations in pathogenic spectrum and antibiotic use provide an important benchmark for further analyses on bacterial genotypes and implementation of antimicrobial stewardship initiatives.

### Limitations

This trial has limitations. First, we used cluster randomization at the whole-NICU level to minimize contamination with health care professionals’ personal perceptions. However, even if cluster-specific characteristics of the randomly allocated clusters are balanced, we had very limited control over the individual participants within each cluster. Furthermore, we did not adjust for infant- or site-level confounders, but we used monthly measures as proxy. Protocol deviations could have occurred on 2 levels: the cluster level, meaning that a center would not have adhered to the allocated order of phases or would have followed only 1 regimen, and at the individual level. A priori, we estimated that the risk for these deviations would be very low and therefore decided to report end points from the ITT dataset. When we excluded data from infants born in clusters with nonadherence, we confirmed noninferiority below our margin delta of 5%.

Second, significant variation in adherence to hygienic guidelines, including glove adherence, may have occurred despite regular training. In addition, extended barrier protection measures were temporarily implemented due to cocolonization with pathogens being resistant to more than 2 groups of antimicrobial agents or *S aureus* and during the COVID-19 pandemic, with different IPC approaches (eg, entry restriction for parents, wearing masks, and proactive surveillance).^26^ However, in a large multicenter study of extremely preterm infants, rates of late-onset sepsis remained unchanged during the pandemic, suggesting no major confounding effects.^27^ We did not assess adherence data on an individual health care professional level due to clinician privacy concerns. For future studies, we suggest to include the number of health care professionals and their self-reports on following hygiene recommendations.

Third, due to pragmatic reasons in a multicenter context, we did not include a formal washout period before crossover. We furthermore assumed that infants from the same site under the same intervention are exchangeable. We actively decided against the alternative assumption that infants in the NICU during the same time window may be more correlated with each other compared to infants across months.

Fourth, we used a relatively large noninferiority margin for sample size calculation. This was based on the assumption that the involved NICUs are highly variable in their spectrum of infants with high risk. Therefore, we considered the individual NICU as a specific ecosystem, with heterogeneous potential for infant-to-infant bacterial transmission, as outlined elsewhere.^7,28,29^ While the number of clusters is high, we were not able to estimate or influence the number of infants at risk of BSI within the cluster. Hence, we expected a wide range of GNB BSI infection rates across sites, which was an overestimation. In addition, we defined transmissions and outbreaks per discretion of the attending study team (ie, bacterial species involved shared identical resistance patterns). In a secondary analysis outside the scope of this report, we will perform whole-genome sequencing of involved bacterial species, which is the most discriminative diagnostic method.^30^ Furthermore, it should be emphasized that our data only apply to routine care in nonoutbreak settings, as outbreaks might still warrant additional precautions.

## Conclusions

This cluster-randomized clinical trial found that standard hand hygiene disinfection for the care of infants colonized with 3GC-GNB was noninferior to standard hygiene disinfection plus extended barrier precautions with regard to GNB BSI rates in the NICU. The BALTIC trial supports a shift toward more targeted, evidence-based IPC practices, allowing resources to be focused where they are most effective.

## Notes

Supplement 1.
Trial Protocol and Statistical Analysis Plan

Supplement 2. **eFigure 1.** Study Design of the BALTIC Trial **eFigure 2.** Center Specific End Points During Study Period **eFigure 3.** Center Specific Colonization And Transmissions

Supplement 3.
Data Sharing Statement
