# Error in Visual Abstract

**DOI:** 10.1001/jamanetworkopen.2026.24497

**Published:** 2026-06-25

In the Original Investigation titled “Extended Barrier Precautions vs Hand Hygiene Alone and Neonatal Sepsis in Intensive Care Patients: The BALTIC Cluster-Randomized Clinical Trial,”^1^ published May 15, 2026, the risk difference in the visual abstract should have been presented as −0.03%; 95% CI, −0.43% to 0.38%. This article has been corrected.^1^
