# Supplementary material for: Extended Barrier Precautions vs Hand Hygiene Alone and Neonatal Sepsis in Intensive Care Patients: The BALTIC Cluster-Randomized Clinical Trial
Source: JAMA Netw Open. 2026 May 15;9(5):e2612759. doi: 10.1001/jamanetworkopen.2026.12759 (PMC13179548; doi:10.1001/jamanetworkopen.2026.12759)
Supplement: Supplement 1. — Trial Protocol and Statistical Analysis Plan [file jamanetwopen-e2612759-s001.pdf]

Study protocol of the cluster-randomized, cross-over, non-inferiority BALTIC trial

Synopsis

Background: There is an urgent need to optimize and evaluate infection prevention and control (IPC) strategies for the care of highly vulnerable infants. Several infection outbreaks in German neonatal intensive care units (NICUs) required rapid responses by hospitals and improved future preparedness. As a consequence, German authorities recommended weekly colonization screening on NICUs. This screening aims to detect multidrug-resistant organisms (MDRO) and bacteria with high transmissibility. According to these guidelines, infants colonized with multiresistant gram-negative (MRGN) bacteria with *in-vitro* resistance to piperacillin and cephalosporins (2MRGN) should be cared wearing non-sterile gloves and gowns in addition to standard hygiene precautions. Whether these extended IPC measures have an individual benefit for infants or contribute to the prevention of infection outbreaks is unknown.

Acronym: BALTIC Barrier protection to lower transmission and infection rates with Gram-negative 2-MRGN in preterm children

Design: Multicenter, cluster-randomized controlled rial with cross over after 12 months

Population: infants requiring neonatal intensive care

Intervention: Focus on hand desinfection for the care of 2MRGN colonized infants in NICUs

Control: Current standard hand desinfection + gloves and gowns (barrier care) for the care of 2MRGN colonized infants in NICUs

Outcome: rate of healthcare-associated (HA) Gram-negative bloodstream infections

Methods: NICUs will be screened for participation and randomly allocated to two trial arms: receiving the intervention "standard precautions with a special focus on hand desinfection" or control (standard precautions "plus" barrier care) for the care of 2MRGN positive infants. Cross over will be performed after 12 months for another 12 months per site. Secondary outcomes included transmission rate with screening relevant bacteria, overall rate of clinical and culture-proven infections, number of antibiotic cycles and desinfectant use. Regular trainings and hygiene audits are standardized co-interventions.

According to our single center data, 9.3% of NICU-treated infants are colonized with 2MRGN during their hospital stay.

Statistical power: We plan with 12 sites in 2 phases (intervention and control phase). Ar least 18 patients per site and phase in 12 sites will be required, thus n=432 in total; accounting for a patient drop-out of maximally 10%, 480 patients need to be included in total. Collecting data over 12 months per phase ensures inclusion of a sufficient number. Estimation of infection risk differences between intervention and control phases will be performed using generalized estimating equations (GEE) models for binary endpoints with identity link; test of non-inferiority with margin of 0.05 using one-sided significance level of 0.05

Conclusions: BALTIC should contribute to better evidence on the effectiveness of hand desinfection and extended barrier precautions in critically ill newborns. Further benefits include comprehensive multi-center data collection on MDRO colonization dynamics, an improved awareness on IPC strategies and establishment of network platforms including antimicrobial stewardship programs.

Background and rationale

Advances in perinatal medicine and neonatology have led to significantly improved survival rates for extremely immature premature babies. Modern care of very low birthweight infants (VLBWI) and critically ill newborns is well standardized in neonatal intensive care units (NICUs). However, the individual immaturity of vulnerable infants and the need for intensive care measures (e.g. vascular catheters, ventilation) are associated with a high risk for healthcare-associated (HA) infections. The overall incidence of bloodstream infection (BSI) in VLBWI ranges from 10-20%, while the rate of Gram-negative sepsis is 3-5% (1). In VLBWI, 20% of intra-hospital deaths are causally linked to HA infections (2, 3), while survivors are susceptible for long-term sequelae, e.g. chronic lung disease as well as neurocognitive and behavioral deficits (4). Hence, there is an urgent need to optimize prevention strategies against HA infections and to provide more evidence on infection control bundles.

The period of highest vulnerability for HA infections is between day 7 and day 28 of life. This critical time frame is characterized by a dynamic colonization with hospital-endemic flora and frequent exposure to antibiotics (5). It has been demonstrated that bacteria colonizing infants might be concordant with invasive pathogens or even starting points of outbreaks (6). Following several infection outbreaks in German NICUs, the German Commission on Hospital Hygiene and Infection Prevention at the Robert-Koch Institute (KRINKO), Berlin, decided to recommend weekly colonization screening with rectal and pharyngeal swabs for infants requiring intensive care. This screening should be performed according to local microbiological standards in order to detect the following pathogens:

KRINKO I – all multi-drug resistant organisms (MDRO), i.e. Multidrug-resistant Gram negative bacteria (MRGN), 2MRGN (resistant to ureidopenicillins, third generation cephalosporins), 3MRGN (resistant to ureidopenicillins, third generation cephalosporins and fluorchinolones)

and 4MRGN (resistant to ureidopenicillins, third generation cephalosporins, fluorchinolones, and carbapenems); methicillin resistant Staphylococcus aureus (MRSA).

KRINKO II – Acinetobacter spp., *Klebsiella pneumoniae*, *Methicillin-susceptible S. aureus (MSSA)*.

KRINKO III – pathogens with high epidemic potential (nosocomial transmissibility) but not MDRO including *Serratia marcescens*, *Pseudomonas aeruginosa*, and Enterobacter spp.

Based on positive screening results proactive hygiene interventions are to be implemented in daily care. This strategy aimes to (a) adjust anti-infective therapy for suspected sepsis in colonized infants, (b) set up an early warning system for outbreaks, (c) prevent spreading of MDRO or pathogens with high epidemic potential by extended barrier precautions and (d) benchmark local epidemiology of MDRO (7). According to KRINKO recommendations, NICU treated infants who are colonized with 2MRGN should be treated with barrier care (protective disposable gloves and long-sleeved gowns with cuffs during any patient contact) in addition to hand desinfection. However, the implementation of these extended hygiene measures should be effective to prevent HA infections and their deleterious consequences which has not yet been clearly shown (3, 8). In addition, side effects of barrier protection need to be considered, e.g. reduced patient contact time, decreased quality of hand disinfection as well as economical and ecological burden of single-use of hygienic items.

The rationale for a cluster-randomized controlled non-inferiority study

The clinical equipoise for this randomized controlled trial (RCT) is based on a potential benefit of extended barrier precautions for reducing HA infection risk and outbreaks in vulnerable babies versus negative effects of potentially non-effective extended hygiene measures. Specifically, a retrospective cohort study including 337 infants suggested that the combined

use of alcohol-based hand rub and gloves leads to a 2.8 fold reduction of HA infections (9). Using a hand hygiene protocol with hand washing, hand rub and gloves a single center study including 200 preterm infants born at 32-36 weeks of gestation reduced HA infections from five to zero cases over a 14-month observation period (10). In a prospective trial including 120 preterm infants, Kaufman (11) noted a 17% reduction of Gram-positive infections and 64% fewer central line-associated bloodstream infections when non-sterile gloves were used as compared to hand disinfection alone. One of the largest efforts in adults, the Benefits of Universal Glove and Gown (BUGG) cluster randomized trial, found non significant effect of universal glove use on preventing infections with MDRO (12). Hence there is a need to evaluate the potential benefits of extended precautions and balance against negative effects. Since the introduction of routine colonization screening in 2013, obervational data from German NICUs revealed no significant changes in infection rates and sepsis mortality caused by pathogens included in the screening (3, 13). While the rate of culture-proven sepsis with Gram-positive bacteria had already been declining in centers participating in a large German cohort study (German neonatal network, GNN) before the screening guideline, the infection rates with Gram-negative pathogens and MRGN remained constant, i.a. 2.5-3%, and < 0.5%, respectively (3). These data provide the key argument for the non-inferiority design of the our trial.

The following drawbacks extended barrier precautions exist. First, glove use can be a key barrier to appropriate hand hygiene (14, 15). In a recent feasibility trial involving 750 infants, Khan et al. (16) found higher hand hygiene compliance rates in standard care time periods as compared to glove use periods at different moments of care: before patient contact or clean procedures as well as after body fluid or direct patient contact. Glove use may therefore confer a false sense of security, as pathogens easily contaminate gloves without performing

hand hygiene properly. Second, our cohort studies demonstrated an overuse of reserve antibiotics such as carbapenems (3, 17) associated with implementation of the weekly colonization screening in Germany. Third, extended hygiene measures are time and cost-intensive and may interfere with other priorities of neurodevelopmental support and family-centered care, e.g. avoidance of co-bedding or skin-to-skin contact of twins with different screening results. Fourth, disposable gloves are the most commonly used single-use item in intensive care. Their production requires natural resources, and they significantly contribute to hospital waste (18). To our best knowledge, there is no evidence on particular benefits on the use of gowns in the care of NICU patients (19).

In the BALTIC trial following hypothesis will be tested:

The care of NICU-treated infants with 2MRGN colonization with standard infection prevention and control stategies (IPC) alone (intervention) is not inferior to standard hand hygiene combined with the use of protective gowns and single-use gloves (control) in terms of the rate of HA infections caused by with Gram-negative pathogens (primary endpoint).

The BALTIC trial can add new data to current uncertainties. The multi-center network structure may generate population-representative data on the prevalence of MRGN in German NICUs. BALTIC also provides the basis for implementing a multi-center antimicrobial stewardship program. We also aim to evaluate the costs of extended hygiene measures given the increased consumption of disposable items.

Benchmarking by analysing single center data

For the design of the Cluster-RCT we evaluated single center data of routine colonization screening (1x/week rectal swab and pharyngeal swab) from VLBWI born between January 1st, 2013 until December 31st, 2017 (n=319 infants, summarized in table 1).

Table 1 Clinical characteristics of single center cohort of very-low-birth weight infants

Clinical parameter		cohort<br>n = 319
Sex, female, n (%)		160 (51)
Multiple birth, n (%)		114 (36)
Gestational age, wk, median (IQR)		28,5 (4,1)
Birth weight, g, median (IQR))		1091 (564)
Mode of birth, n (%)	Spontaneous	20 (6)
	Elective casearean	256 (80)
	Emergency Caesarean	43 (14)
Cause of preterm birth, n (%)<br>Multiple causes possible	Preterm labor	194 (44)
	Pre-eclampsia	39 (9)
	Pathological Doppler	112 (25)
	Placental abruption	4 (1)
	Intrauterine growth restriction	63 (20)
	Suspected or proven amniotic infection	144 (45)
Duration hospital stay, days, median (IQR)		63 (41)
Antibiotic days/100 days, median (IQR)		13,1 (14,9)
Probiotic use, n (%)		174 (55)

Out of 319 infants, 47 (14.7%) were colonized with an MRGN pathogen, of which 30 infants (9.3%) tested positive for a 2MRGN pathogen. One infant was colonized with 2MRGN *E. coli* at birth, 15 infants were colonized within the first 5 weeks of life (figure 1). The 2MRGN pathogens were isolated from pharyngeal swabs (n=12), rectal swabs or fecal samples (n=19) or conjunctiva (n=1). Four infants were colonized with > 1 MDRO including two infants with MRSA and 3MRGN *E.coli* and two infants with 2MRGN *Enterobacter cloacae* and either 2MRGN *E.coli* or 2MRGN *Klebsiella pneumoniae*. Colonization with 2MRGN occurred at a median of 33 days. Figure 1 describes the timepoint of first colonization with MDRO in vulnerable infants. Seven infants were initially colonized with antibiotic susceptible bacteria and acquired 2MRGN features during hospital stay.

Out of 319 of infants, 296 received antibiotic therapy for a median duration of 7 days, 252 infants were already started on antibiotics on day 1 of life. MDRO colonized infants were treated for a median of 7 days. Among 2MRGN colonized infants, one infant developed a 2MRGN *E.coli* sepsis on day 6 of life, two infants were diagnosed with 2MRGN sepsis (*Enterobacter* spp.) between day 7 and 28 of life and five infants were treated for culture-negative sepsis. Our data are in line with previous single center studies in Germany with evidence of MDRO colonizing infants in 4.9% of 635 neonates, 12.7% of 584 screened neonates and 15.3% of 1407 preterm infants, respectively (20-22). In a recent multicenter RCT on the effect of probiotics to prevent MDRO colonization in preterm infants 28-32 weeks of gestation, we confimed center-specific MDRO colonizing rates between 5-20% (23). In the BALTIC trial, we therefore estimated the number of 2MRGN colonized infants to be 10% during the hospital stay.

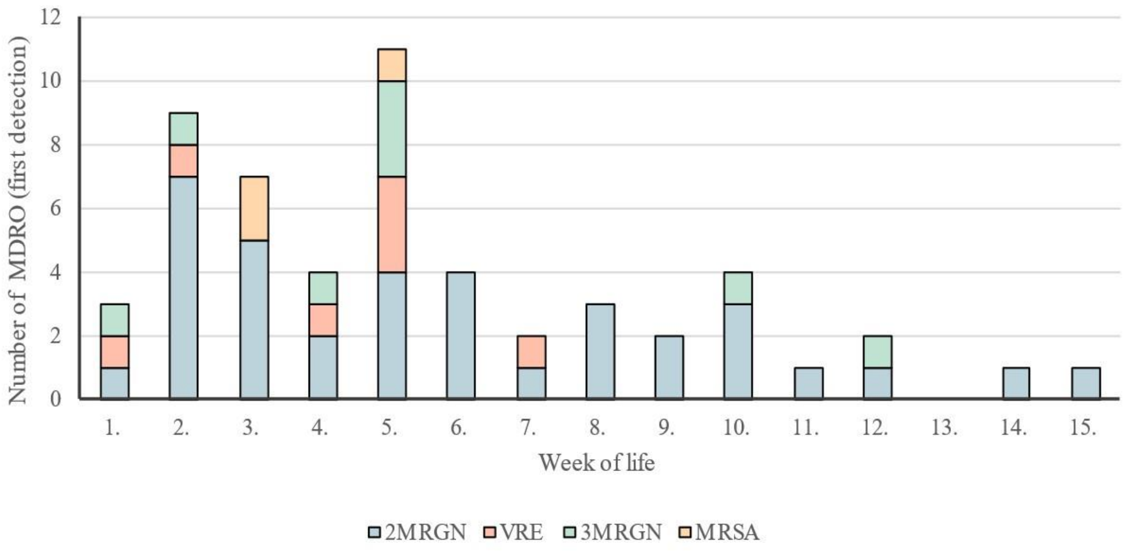

Figure 1: First detection of MRGN, MRSA, MRSA or Vancomycin resistant enterococci (VRE)

MRDO = Multi-drug resistant organisms; KRINKO = German Commission on Hospital Hygiene and Infection Prevention, MRGN = Multidrug-resistant Gram negative bacteria, 2MRGN (resistant to Piperacillin and Cefotaxim or Ceftazidim), 3MRGN (resistant to Piperacillin and Cefotaxim or Ceftazidim and fluorchinolones), MRSA = methicillin-resistant *Staphylococcus aureus*, VRE = Vancomycin resistant *Enterococci*

Additional first detections after the 15th week of life: n = 4 2MRGN pathogens

Trial design

In the participating centers, all newborns requiring intensive care undergo colonization screening based on KRINKO recommendations. If a 2MRGN positive pathogen is detected this infant is treated with hygiene measures as described in the intervention (focus on standard hand disinfection) or control procedure (standard hand disinfection plus barrier precautions), depending on the randomization of the center. The study has been conducted in a cross-over design with a change between the intervention and control group after 12 months**.** All other patients (screening negative or positive for a pathogen included in the KRINKO recommendations apart from 2MRGN) receive the standard care based on screening results and local guideline. After 6, 12, 18 and 24 months, the compliance will be evaluated by the means of random observations of hygiene in the NICU in collaboration with local institutions for hospital hygiene followed by standardized training modules. The PICO question of this multicenter, cluster RCT with cross over is whether in the **P**opulation of preterm infants and critically ill neonates requiring intensive care who are colonized with 2MRGN the **I**ntervention ***standard hand hygiene disinfection*** is non-inferior to the current KRINKO recommendation (**C**ontrol) of ***standard hand hygiene disinfection + barrier precautions (gloves/gown)*** for the **O**utcome overall rate of HA Gram-negative bloodstream infections (primary endpoint).

Site selection and randomization

Neonatal intensive care units with level 1 care will be included in the study. Randomization will be performed at the level of the individual center (computerized sequence tool), and sites will be randomly allocated to start with either intervention or control group with after on-site training and audit in hygiene measures. For site selection, we synergistically use our collaborative networks of the GNN and the PRIMAL study which proved reliability in previous

clinical studies and enables to compare data on infections before during the BALTIC trial. Background information about the participating sites are collected through a structured interview, followed by a baseline observation, hand hygiene training and audit. Hand hygiene training will include presentations on hand hygiene compliance in NICUs and room for improvement, guidelines on good hand hygiene practice and other measures of infection control, consequences of HA infections and outbreaks, distribution of flyers/posters related to the WHO's "5 Moments forHand Hygiene" (24) and access to videos/webinars/e-learning. The audits and training will be organized by the clinical project management team in Lübeck, in collaboration with the respective hospital hygiene leadership and the hygiene officers (Link Nurses) / physicians.

Further requirements for participation in the study are (a) implementation of an admission screening and a weekly colonization screening as recommended, (b) availability of a microbiological laboratory that collects antimicrobial resistance data and supports further measures in the event of a suspected HA infection outbreak, (c) support from the local institutions for hospital hygiene for hygiene training and audits and (d) support from the local pharmacies and health business departments which provide data on antibiotic consumption and gown/glove use.

Audits

Quality controls and data security are ensured by the availability of a study protocol, by a monitoring visit prior to the start of the study and by standard operating procedures (SOP, e. g. for diagnosis of sepsis, transmission). Audits are initiated to verify adherence to the study protocol and ICH-GCP (Good clinical practice) procedures.

In order to evaluate the compliance with infection control measures, direct observations are planned on a random basis by the study team and IPC nurses. The observations will not affect

other treatment priorities, e.g. kangaroo care. We aim for anonymous documentation of disinfectant use (nurse, doctor, service staff, parents, others), and compliance with 5 moments of proper hand hygiene, use of gloves and long-sleeved gowns. The Hawthorne effect, i.e. alteration of typical behavior by being observed and variability among observers (external representatives of the study leadership, local hygiene experts, and link nurses) is a potential disadavantage of the direct observation approach (25). We therefore collect data on disinfectant use in the clusters (mL per patient day) which serves as a surrogate for hygiene compliance.

Management of transmissions and possible outbreaks will be at the discretion of the local team based on NICU guidelines which was different across the NICUs. The local standards are documented at initiation of the study-centers, at after 12 months (cross-over) and at the end of the study after 24 months. In the manuscript reporting data we will also report on extended hygiene measures and additional tests for outbreak identification (e.g., genotyping, pulse field electrophoresis)

Outcomes

The primary endpoint is the rate of HA bloodstream infections with Gram-negative pathogens in all preterm and newborn infants who received colonization screening. This outcome is defined clinically according to NEO-KISS criteria (at least two clinical signs of sepsis + one laboratory sign, e.g. increase in C-reactive protein > 10mg/l) after 72 hours of life and requires the detection of a Gram-negative pathogen in the blood culture. Blood-culture isolates will be sent from the study centers to the study management.

Key secondary endpoint is the 2MRGN transmission rate which is assumed if another infant is colonized with the same 2MRGN pathogen after 2MRGN colonization or infection of an infant

requiring intensive care in a temporal (maximum 90 days) and spatial context (potential contact in the intensive care unit) (26). This assumption is made if, in addition to pathogen identification, the antimicrobial susceptibility testing patterns are identical. Suspected transmission of or bloodstream infection with an 2MRGN pathogen will be reported to the study management as severe adverse event within 24 hours.

Transmission/outbreak management remains the responsibility of the local interdisciplinary infection control team and is carried out according to local standards, including the decision whether to initiate genetic identity testing in the acute situation. 2MRGN isolates will be shipped from local sites and molecular typing will be performed at the Departemnt of Infectious Diseases and Medical Microbiology at the University of Lübeck. Other secondary outcomes at the level of study sites per period ("study phase") are:

- Rate of clinical and culture proven infections
- Rate of infection outbreaks
- Number of initiated antibiotic cycles
- Duration of antibiotic treatment (days)
- Exposure to reserve antibiotics, including Carbapenems per study site in % infants.

To assure documentation of relevant outcomes, intensive interdisciplinary collaboration (neonatology/infectiology/hygiene/pharmacy/management) is required. Data are collected by monthly cluster report forms on infection and transmission rates and yearly surveillance of antibiotic use, disinfectant consumption, and cost estimations.

Stopping rules

The coordinating investigator has the right to discontinue the study in centers that experience insufficient study conduction, technical-logistic problems and repetitive outbreak situations that require extended barrier precautions in all patients, which would interfere with the study

questions. The study sites will be instructed to discontinue the study only if substantial problems occur. The coordinating investigator will be informed immediately, should ethical or safety concerns occur at any study site.

Quality assurance and monitoring

During the study, quality assurance measures including supervision by authorities will be implemented. All study centers have agreed that a study monitor will review the data collection before, during and after the study to ensure that the study is conducted in accordance with the protocol, with SOPs and the corresponding regulations of GCP. In addition to the monitoring procedures, audits will be carried out in accordance with the ICH-GCP Guidelines. In the context of an audit, the planning, execution, and analysis of the study are checked to evaluate whether all study processes are conducted in accordance with the German law or the ICH Guidelines. This includes the storage of data and the organisation of the study centres, the original documents in accordance with data management agreements between sponsors and participating centres. The objective of audit measures is to ensure that all results and conclusions that ultimately appear in the final report are readable from the raw data.

Data management

The primary data set is generated and managed by the clinical project management of the study management. The data generated on the basis of the CRFs are entered into the central database at the study management using the "double-entry" principle. To ensure optimal data protection, the data on settlement or primary endpoints are stored and evaluated without any reference to the name of the child or the personal data of the parents. Pathogen isolates are stored without patient identifying data but date of collection to allow a temporal and

cluster-related assignment (retrospective detection of transmission by central biobanking and pathogen genotyping). The provisions of the Data Protection Act are strictly adhered to, ensuring the data is safeguarded from third-party access.

Sample size calculation and statistical analysis

GNN and PRIMAL centers treat on average 150 preterm and critically ill neonates per year with compulsory colonization screening. The center-specific rates of Gram-negative HA infection ranges between 1.5 and 6%. Based on single center data as described above and other cohort studies (20-22) we expect 10% of these infants to be colonized with 2MRGN during hospital stay, i.e. 180 infants per 12 months observational period.

Based on previous data, we assume an intracluster correlation of maximally 0.01. Given that the observed correlation is very low, the time lag between the two trial periods is long and different patients are included in the two trial periods at each site. No carry-over effects are assumed, as we are not expecting that effects of one treatment period would influence the second period (after cross-over) since medical care-givers are aware of the study period they are in. Date from two periods at one site are regarded as two independent sites. We further assume a frequency of Gram-negative HA infections in the controls of 3% and a non-inferiority limit of 5%. Striving for a power of 0.8 (1-ß) with a one-sided significance level α of 0.05, at least n=18 patients per site and phase are required for analysis with 12 sites, thus n=432 in total. To allow for a maximal drop-out of 10% drop-out, at least 480 patients need to be included.

The primary data analysis will be performed according to the intention-to-treat principle (non-linear mixed effect regression models, logit link). Generalized estimating equations are performed to determine the infection rates between the two regimens. Secondary endpoints are analysed as the primary endpoint per individual or per cluster.

Data dissemination

After completion of the study, the results will be presented at national and international congresses and published in peer-reviewed journals. The results are also presented in the media of the parents' associations (Federal Association "The Pregnant Child" and EFCNI). The public is informed about the study in the form of press releases and background articles on preterm birth and infection risks.

Funding

The study is partially funded by the German Ministry of Education and Research (German Center for Infection Research), the German Society for Pediatric Infectious Diseases and the Damp Stiftung.

Perspectives

The BALTIC study was drafted to create evidence on the effectiveness of extended barrier precaution measures in critically ill newborns being colonized with MDRO. Current guidelines recommend regular screening and the consequent use of gloves and gowns in case of 2MRGN colonization, however, the evidence on clinically relevant outcomes is scarce. Potential disadvantages of extended barrier precautions are time- and cost intensity and may interfere with individualized neurodevelopmental care. The multicenter design of BALTIC creates new insights into center-specific endemic flora of NICUs and population-representative data on MDRO colonization dynamics. Standardized trainings on the proper use of infection control measures will lead to an improved awareness on preventive strategies and the establishment of network platforms including antibiotic stewardship programs. This study also has

limitations. Training and audits will be performed as controlled co-intervention, however, significant variation in compliance due to Hawthorne or observer bias and irregular attendance of healthcare professionals for training lessons cannot be prevented. Considering that there are few studies that have rigorously investigated the current recommendations for infection control in neonatology, BALTIC will contribute to more refined and successful interventions in the future.

References

1. Humberg, A., Härtel, C., Rausch, T. K., Stichtenoth, G., Jung, P., Wieg, C., Kribs, A., von der Wense, A., Weller, U., Höhn, T., Olbertz, D. M., Felderhoff-Müser, U., Rossi, R., Teig, N., Heitmann, F., Schmidtke, S., Bohnhorst, B., Vochem, M., Segerer, H., Möller, J., Eichhorn, J. G., Wintgens, J., Böttger, R., Hubert, M., Dördelmann, M., Hillebrand, G., Roll, C., Jensen, R., Zemlin, M., Mögel, M., Werner, C., Schäfer, S., Schaible, T., Franz, A., Heldmann, M., Ehlers, S., Kannt, O., Orlikowsky, T., Gerleve, H., Schneider, K., Haase, R., Böckenholt, K., Linnemann, K., Herting, E., and Göpel, W. (2020) Active perinatal care of preterm infants in the German Neonatal Network. *Archives of disease in childhood. Fetal and neonatal edition* 105, 190-195.

2. Flannery, D. D., Chiotos, K., Gerber, J. S., and Puopolo, K. M. (2022) Neonatal multidrug-resistant gram-negative infection: epidemiology, mechanisms of resistance, and management. *Pediatric research* 91, 380-391.

3. Härtel, C., Faust, K., Fortmann, I., Humberg, A., Pagel, J., Haug, C., Kühl, R., Bohnhorst, B., Pirr, S., Viemann, D., Simon, A., Zemlin, M., Poralla, S., Müller, A., Köstlin-Gille, N., Gille, C., Heckmann, M., Rupp, J., Herting, E., and Göpel, W. (2020) Sepsis related mortality of extremely low gestational age newborns after the introduction of colonization screening for multi-drug resistant organisms. *Antimicrobial resistance and infection control* 9, 144.

4. Mukhopadhyay, S., Puopolo, K. M., Hansen, N. I., Lorch, S. A., DeMauro, S. B., Greenberg, R. G., Cotten, C. M., Sanchez, P. J., Bell, E. F., Eichenwald, E. C., and Stoll, B. J. (2021) Neurodevelopmental outcomes following neonatal late-onset sepsis and blood culture-negative conditions. *Archives of disease in childhood. Fetal and neonatal edition* 106, 467-473.

5. Schwartz, D. J., Shalon, N., Wardenburg, K., DeVeaux, A., Wallace, M. A., Hall-Moore, C., Ndao, I. M., Sullivan, J. E., Radmacher, P., Escobedo, M., Burnham, C. D., Warner, B. B.,

Tarr, P. I., and Dantas, G. (2023) Gut pathogen colonization precedes bloodstream infection in the neonatal intensive care unit. *Science translational medicine* 15, eadg5562.

6. Folgori, L., Tersigni, C., Hsia, Y., Kortsalioudaki, C., Heath, P., Sharland, M., and Bielicki, J. (2018) The relationship between Gram-negative colonization and bloodstream infections in neonates: a systematic review and meta-analysis. *Clinical microbiology and infection : the official publication of the European Society of Clinical Microbiology and Infectious Diseases* 24, 251-257.

7. KRINKO (2013) Praktische Umsetzung sowie krankenhaushygienische und infektionspräventive Konsequenzen des mikrobiellen Kolonisationsscreenings bei intensivmedizinisch behandelten Früh- und Neugeborenen - Ergänzende Empfehlung der KRINKO beim Robert Koch-Institut, Berlin, zur Implementierung der Empfehlungen zur Prävention nosokomialer Infektionen bei neonatologischen Intensivpflegepatienten mit einem Geburtsgewicht unter 1500g aus dem Jahr 2007 und 2012. *Epidemiologisches Bulletin* 42, 422-431.

8. Seidel, J., Haller, S., Eckmanns, T., and Harder, T. (2018) Routine screening for colonization by Gram-negative bacteria in neonates at intensive care units for the prediction of sepsis: systematic review and meta-analysis. *The Journal of hospital infection* 99, 367-380.

9. Ng, P. C., Wong, H. L., Lyon, D. J., So, K. W., Liu, F., Lam, R. K., Wong, E., Cheng, A. F., and Fok, T. F. (2004) Combined use of alcohol hand rub and gloves reduces the incidence of late onset infection in very low birthweight infants. *Archives of disease in childhood. Fetal and neonatal edition* 89, F336-340.

10. Janota, J., Šebková, S., Višňovská, M., Kudláčková, J., Hamplová, D., and Zach, J. (2014) Hand hygiene with alcohol hand rub and gloves reduces the incidence of late onset sepsis in preterm neonates. *Acta Paediatr* 103, 1053-1056.

11. Kaufman, D. A., Blackman, A., Conaway, M. R., and Sinkin, R. A. (2014) Nonsterile glove use in addition to hand hygiene to prevent late-onset infection in preterm infants: randomized clinical trial. *JAMA pediatrics* 168, 909-916.

12. Harris, A. D., Morgan, D. J., Pineles, L., Magder, L., O'Hara, L. M., and Johnson, J. K. (2021) Acquisition of Antibiotic-Resistant Gram-negative Bacteria in the Benefits of Universal Glove and Gown (BUGG) Cluster Randomized Trial. *Clinical infectious diseases : an official publication of the Infectious Diseases Society of America* 72, 431-437.

13. Litz, J. E., Goedicke-Fritz, S., Härtel, C., Zemlin, M., and Simon, A. (2019) Management of early- and late-onset sepsis: results from a survey in 80 German NICUs. *Infection* 47, 557-564.

14. Pittet, D., Hugonnet, S., Harbarth, S., Mourouga, P., Sauvan, V., Touveneau, S., and Perneger, T. V. (2000) Effectiveness of a hospital-wide programme to improve compliance with hand hygiene. Infection Control Programme. *Lancet* 356, 1307-1312.

15. Fuller, C., Savage, J., Besser, S., Hayward, A., Cookson, B., Cooper, B., and Stone, S. (2011) "The dirty hand in the latex glove": a study of hand hygiene compliance when gloves are worn. *Infection control and hospital epidemiology* 32, 1194-1199.

16. Khan, S., Tsang, K. K., Hu, Z. J., Mostowiak, B., El Helou, S., Science, M., Kaufman, D., Pernica, J., Thabane, L., Mertz, D., and Loeb, M. (2023) GloveCare: a pilot study in preparation for a cluster crossover randomized controlled trial of non-sterile glove-based care in preventing late-onset infection in the NICU. *Pilot and feasibility studies* 9, 50.

17. Härtel, C., Hartz, A., Bahr, L., Gille, C., Gortner, L., Simon, A., Orlikowsky, T., Müller, A., Körner, T., Henneke, P., Haase, R., Zemlin, M., Viemann, D., Gebauer, C., Thome, U., Ziegler, A., Rupp, J., Herting, E., and Göpel, W. (2016) Media Stories on NICU Outbreaks Lead to an

Increased Prescription Rate of Third-Line Antibiotics in the Community of Neonatal Care. *Infection control and hospital epidemiology* 37, 924-930.

18. Hunfeld, N., Diehl, J. C., Timmermann, M., van Exter, P., Bouwens, J., Browne-Wilkinson, S., de Planque, N., and Gommers, D. (2023) Circular material flow in the intensive care unit-environmental effects and identification of hotspots. *Intensive care medicine* 49, 65-74.

19. Alslaim, H. S., Chan, J., Saleem-Rasheed, F., Ibrahim, Y., Karabon, P., and Novotny, N. (2022) Discordance among Belief, Practice, and the Literature in Infection Prevention in the NICU. *Children (Basel, Switzerland)* 9.

20. Haase, R., Worlitzsch, D., Schmidt, F., Kulka, R., Kekulé, A. S., and Körholz, D. (2014) Colonization and infection due to multi-resistant bacteria in neonates: a single center analysis. *Klinische Padiatrie* 226, 8-12.

21. Baier, C., Pirr, S., Ziesing, S., Ebadi, E., Hansen, G., Bohnhorst, B., and Bange, F. C. (2019) Prospective surveillance of bacterial colonization and primary sepsis: findings of a tertiary neonatal intensive and intermediate care unit. *The Journal of hospital infection* 102, 325-331.

22. Bubser, C., Liese, J., Serna-Higuita, L. M., Müller, A., Vochem, M., Arand, J., Karck, U., Gross, M., Poets, C. F., Härtel, C., Zemlin, M., Gille, C., and Köstlin-Gille, N. (2022) Impact of early antibiotic exposure on the risk of colonization with potential pathogens in very preterm infants: a retrospective cohort analysis. *Antimicrobial resistance and infection control* 11, 72.

23. Van Rossum, T., Haiß, A., Knoll, R. L., Marißen, J., Podlesny, D., Pagel, J., Bleskina, M., Vens, M., Fortmann, I., Siller, B., Ricklefs, I., Klopp, J., Hilbert, K., Meyer, C., Thielemann, R., Goedicke-Fritz, S., Kuntz, M., Wieg, C., Teig, N., Körner, T., Kribs, A., Hudalla, H., Knuf, M., Stein, A., Gille, C., Bagci, S., Dohle, F., Proquitté, H., Olbertz, D. M., Schmidt, E., Koch, L., Pirr,

S., Rupp, J., Spiegler, J., Kopp, M. V., Göpel, W., Herting, E., Forslund, S. K., Viemann, D., Zemlin, M., Bork, P., Gehring, S., König, I. R., Henneke, P., and Härtel, C. (2024) Bifidobacterium and Lactobacillus Probiotics and Gut Dysbiosis in Preterm Infants: The PRIMAL Randomized Clinical Trial. *JAMA pediatrics*.

24. WHO, https://cdn.who.int/media/docs/default-source/integrated-health-services-(ihs)/infection-prevention-and-control/your-5-moments-for-hand-hygiene-poster.pdf?sfvrsn=83e2fb0e_21.

25. Wu, K. S., Lee, S. S., Chen, J. K., Chen, Y. S., Tsai, H. C., Chen, Y. J., Huang, Y. H., and Lin, H. S. (2018) Identifying heterogeneity in the Hawthorne effect on hand hygiene observation: a cohort study of overtly and covertly observed results. *BMC infectious diseases* 18, 369.

26. Härtel, C., Faust, K., Avenarius, S., Bohnhorst, B., Emeis, M., Gebauer, C., Groneck, P., Heitmann, F., Hoehn, T., Hubert, M., Kribs, A., Küster, H., Laux, R., Mögel, M., Müller, D., Olbertz, D., Roll, C., Siegel, J., Stein, A., Vochem, M., Weller, U., von der Wense, A., Wieg, C., Wintgens, J., Hemmelmann, C., Simon, A., Herting, E., and Göpel, W. (2012) Epidemic microclusters of blood-culture proven sepsis in very-low-birth weight infants: experience of the German Neonatal Network. *PloS one* 7, e38304.

DECLARATIONS

Statement of Ethics: The BALTIC study was approved by the institutional review board of the University of Lübeck (primary vote, 19-275) and by the review boards of all participating sites (secondary vote). No identifying data about the infants were collected.

Consent for publication: Not applicable

Availability of data and material: The full protocol is registrated in the German registry of clinical studies (DRKS, Trial registration number: DRKS00019103), we plan to grant access to the full dataset.The datasets used and/or analysed for the benchmarking results are available from the corresponding author on reasonable request.

Competing interests: JP received a grant from the German ministry of education and research (#01EO2106). MK has received honoraria for consulting, presentations, expert testimony, and advisory board participation from Abbott, GSK, Pfizer, and Sanofi outside the submitted work. He is member in the board of the AWMF-guidelines "sustainability in intensive care and emergency medicine" and "invasive ventilation and use of extracorporeal oxygenation in patients with respiratory failure" outside the submitted work and has received travel support from the Deutsche Gesellschaft für Hygiene und Mikrobiologie.

The other authors have no conflicts of interest relevant to this article to disclose.

Funding Sources: The study was funded by the German Center for Infection Research, the German Society for Pediatric Infectious Diseases and the Damp Stiftung in 2019. The funders had no role in the design, data collection, data analysis, and reporting of this study.

Author Contributions:

ChH conceptualized and designed the study and drafted the manuscript.

KF supervised and coordinated the study, designed the data collection instruments and critically reviewed and revised the manuscript for important intellectual content.

ClH and JP designed and carried out the analyses of the benchmarking single center clinical data.

JR, EH and AS conceptualized and designed the study and critically reviewed and revised the manuscript for important intellectual content.

MK and ML carried out the quality control (audits and monitoring of the study sites).

IK designed the statistical instruments and carried out th sample size calculation.

RJ, AS, UFM, DF, KL, CW, GH, BN, ES, LK, SS, MZ, SM, CS, NKG, CG, AL, MK and SK coordinated and supervised the inclusion of the different participating study centers and the respective data collection in the study centers.

DN and FS designed the microbiological analyses of the collected pathogens and coordinated the collection.

WG critically reviewed and revised the manuscript for important intellectual content.

All authors approved the final manuscript as submitted and agree to be accountable for all aspects of the work.

Statistical Analysis Plan (SAP)

Barrier protection to **l**ower **t**ransmission and **i**nfection rates with Gram-negative bacteria in preterm **c**hildren

BALTIC

P24069

Document version: Draft V01D01, final version V1.0

Version date: 25.02.2023

Protocol version/date: version 1.0 from 17.07.2019

Signature Page

The signing persons have read and revised the present statistical analysis plan for the study BALTIC and agree to its content.

Sponsor

UKSH, Campus Lübeck, vertreten durch die Klinik für Kinder- und Jugendmedizin (Prof. Egbert Herting; egbert.herting@uksh.de); Ratzeburger Allee 160, 23562 Lübeck, Germany

Studienleiter

Prof. Dr. Christoph Härtel

haertel_c1@ukw.de

Place, date Signature

Principal Investigator

UKSH, Campus Lübeck, vertreten durch die Klinik für Kinder- und Jugendmedizin

Ratzeburger Allee 160, 23562 Lübeck, Germany

Prof. Dr. Christoph Härtel

haertel_c1@ukw.de

Place, date Signature

Biostatisticians

Institut für Medizinische Biometrie und Statistik

Universität zu Lübeck

Ratzeburger Allee 160, Haus 24, 23562 Lübeck

Prof. Dr. Inke R. König

inke.koenig@uni-luebeck.de

Place, date | Signature study biostatistician

[Dr. Maren Vens]

[m.vens@uni-luebeck.de]

Place, date | Signature independent biostatistician

1 LIST OF ABBREVIATIONS 28

2 AIM OF THE STATISTICAL ANALYSIS PLAN 28

3 RESPONSIBILITIES 29

4 STUDY DESIGN AND AMENDMENTS 29

4.1 STUDY 29

5 DATABASE 31

5.1 INCOMPLETE TREATMENTS, DROP-OUTS, AND INCOMPLETE OBSERVATIONS 32
5.2 PROTOCOL DEVIATIONS 32

6 ANALYSIS TIMING 33

7 SETS OF PATIENTS 33

8 HANDLING OF MISSING VALUES, MISSING DATA, AND OUTLIERS 33

8.1 MISSING VALUES, AND MISSING DATA 33
8.2 OUTLIERS AND IMPLAUSIBLE ENTRIES 33

9 STATISTICAL ANALYSES/METHODS 34

9.1 BLINDED DATA REVIEW MEETING 34
9.2 SUBJECT DISPOSITION 34
9.3 ANALYSIS OF PRIMARY OUTCOME VARIABLES 34
9.4 ANALYSIS OF SECONDARY OUTCOME VARIABLES 35
9.4.1 Efficacy Outcome Variables on the level of months 36
9.4.2 Efficacy Outcome Variables on the level of patients 37
9.4.3 Safety/Tolerability Outcome Variables 38

10 SOFTWARE 38

1 List of Abbreviations

Abbreviation	Description
AE	Adverse Event
BSI	Bloodstream Infection
CO	Control group with hand disinfection plus gloves and gowns
CONSORT	Consolidated Standards of Reporting Trials
CRF	Case Report Form
GEE	Generalized Estimating Equations
IMBS	Institute of Medical Biometry and Statistics, University of Lübeck, University Hospital Schleswig-Holstein, Campus Lübeck
ITT	Intention-To-Treat
IV	Intervention group with hand disinfection alone
MRGN	Multi-Resistant Gram-Negative
n.a.	not applicable
NICU	Neonatal Intensive Care Unit
R	R Software
SAP	Statistical Analysis Plan
SOP	Standard Operating Procedures

2 Aim of the Statistical Analysis Plan

Central assumptions, methods, and procedures for the statistical analysis are described in the study protocol. The statistical analysis plan (SAP) specifies in detail the statistical and biostatistical approaches and procedures to be used.

The SAP only includes the statistical analyses for which the Institute for Medical Biometry and Statistics, University of Lübeck, University Hospital Schleswig-Holstein, Campus Lübeck (IMBS) is responsible.

The reference documents of the SAP are

- the protocol of 17.07.2019, version 1,

- the publication of the study protocol (Faust et al. Mol Cell Pediatr 2025)

3 Responsibilities

For data entry, a central Access database was programmed by the data management of the IMBS according to its standard operating procedures (SOPs).

The primary data set is generated and managed by the clinical project management of the study management. The data generated on the basis of the case report forms (CRFs) are entered into the central database at the study management using the "double-entry" principle. To ensure optimal data protection, the data on settlement or primary endpoints are stored and evaluated without any reference to the name of the child or the personal data of the parents.

For the final analysis, the clinical project management team exports the data and transfers it to the biostatistics team of the IMBS.

Statistical analyses will be carried out by the IMBS, independently of the sponsor's/sponsor's representative interests.

Contact Programmer:

Institut für Medizinische Biometrie und Statistik

Universität zu Lübeck

Ratzeburger Allee 160, Haus 24, 23562 Lübeck

Programmer

Frank Sandig

frank.sandig@uni-luebeck.de

4 Study Design and Amendments

4.1 Study

Full title of the study	Barrier protection to lower transmission and infection rates with gram-negative bacteria in preterm children
Short title of the study	BALTIC

Indication	Infection with multiresistant gram-negative (MRGN) bacteria with in-vitro resistance to piperacillin and 3rd generation cephalosporins (2MRGN)
Primary objectives	To show non-inferiority of hand disinfection as compared to hand disinfection + gloves and gowns for the care of 2MRGN colonized infants in neonatal intensive care units (NICUs) with regard to gram negative infections
Secondary objectives	To explore non-inferiority with regard to other transmissions and infections on patient- and month-level
Study design	Cluster-randomized, cross-over, two-arm, multicentric, open
Randomization procedure	Randomization of sequence of interventions for every site, i.e. randomization of clusters stratified by site, by a computerized simple sequence tool
Number of participating sites	12 with 2 phases (intervention and control) each
Planned sample size	At least 18 patients per site and phase in 12 sites, thus n=432 in total; accounting for a patient drop-out of maximally 10%, 480 patients need to be included in total. Collecting data over 12 months per phase ensures inclusion of a sufficient number.
Study intervention	Hand disinfection alone (intervention, IV) versus hand disinfection plus gloves and gowns (control, CO)
Study population	Participation criteria defined for sites and patients, see study protocol
Primary endpoints	Gram-negative infection in newborn infants requiring intensive care and colonization screening
Secondary endpoints	Patient-level endpoints:<br>• any relevant transmission<br>• transmission with 2MRGN<br>• any bloodstream infection (BSI)<br>• 2MRGN BSI<br>• clinical infection<br>Month-level endpoints:<br>• at least one transmission<br>• at least one transmission with 2MRGN<br>• started antibiotics cycles<br>• percentage of days with antibiotics

	• at least one started reserve antibiotic
Statistical analyses	Estimation of infection risk differences between IV and CO using generalized estimating equations (GEE) models for binary endpoints with identity link; test of non-inferiority with margin of 0.05 using one-sided significance level of 0.05
Registration of the study	German Trial Register DRKS00019103

The study was approved by the institutional review board of the University of Lübeck (primary vote, 19-275) and by the review boards of all participating sites (secondary vote).

5 Database

The database is the data taken over from the clinical project management team according to Chapter 3. In this, data is documented entirely on an aggregated level, i.e., per month per site.

The data is structured as follows:

- Every of the 12 sites participates in the study in two phases, in one of which hand disinfection alone (IV), in the other hand disinfection plus gloves and gowns is applied (CO). The order of the two phases is randomized.
- Every phase has a duration of 12 months, so that for every site, data is collected for 24 months.
- Each of the two phases at one site is considered one cluster.
- Given the aggregation of the data in months, the observational unit is the month (month-level variables).
- Some variables give the count of patients with a specific event. From this and the number of admissions in that month, the number of patients without the specific events is constructed as well as data on patient-level is constructed for analyses (patient-level variables).

To obtain the count of patients without a specific event, the number of events is substracted from the number of admissions [`Aufnahmen_Neo`] rendering the following additional variables:

Count of patients with the event (original variable)	Count of patients without the event (constructed variable)
`Anzahl_gramNeg_nurLOS`	`Anzahl_gramNeg_nurLOS_negativ`
`Transm_Patient`	`Transm_Patient_negativ`
`Transmission_2MRGN_Patient_SAE`	`Transmission_2MRGN_Patient_SAE_negativ`
`Anzahl_kl_Sepsis_allg`	`Anzahl_kl_Sepsis_allg_negativ`
`Anzahl_BKpos_Sepsis_allg`	`Anzahl_BKpos_Sepsis_allg_negativ`
`Anzahl_2MRGN_LOS`	`Anzahl_2MRGN_LOS_negativ`

To allow for patient-level description, the data set is re-structured to contain one line per patient based on the documented number of patients admitted to the site [Aufnahmen_Neo]. In this, the following month-level variables are used to construct the following patient-level variables indicating presence or absence of the event:

Variable	Month-level (original variable)	Patient-level (constructed variable)
Gram negative infection	Anzahl_gramNeg_nurLOS	gramNeg_nurLOS
At least one suspected transmission	Transm_Patient	Transm
At least one suspected transmission of 2MRGN	Transmission_2MRGN_ Patient_SAE	Transm2m
Clinical infection	Anzahl_kl_Sepsis_allg	Sepsis
Any BSI	Anzahl_BKpos_Sepsis_allg	Sepsis_pos
2MRGN BSI	Anzahl_2MRGN_LOS	LOS

For the estimation of the GEE models, a cluster variable [Cluster] is created that combines the information from the site identifier [Klinik_ID] with the variable indicating the phase [Randomisierung], so that every site corresponds to two clusters, one for every phase.

The variable counting the number started reserve antibiotics in a month [Anzahl_beg_Reserve_ATB] is dichotomized to indicate whether at least one treatment with a reserve antibiotic was started or not [Beg_Reserve_ATB].

In addition, the clinical project management team surveys the participating sites about consumption of disinfectant and estimated costs. This data will separately be transferred to the IMBS for analysis.

5.1 Incomplete Treatments, Drop-Outs, and Incomplete Observations

Data is documented entirely on an aggregated level, i.e., per month per site. Therefore, there is no documentation on missing data on the individual level. Missing entries at the aggregated level are possible, and for every analysis, the number of valid entries will be clearly stated.

Reporting and visualization comply with the CONSORT guideline in its current version.

5.2 Protocol Deviations

For every month, the percentage of patients with 2MRGN infection who are not treated according to randomization is documented [Nicht_nach_Rando_Proz] and reported.

No further protocol deviations are recorded.

6 Analysis Timing

No interim analyses are planned. The final analysis is performed after database closure and export to the IMBS.

7 Sets of Patients

Inclusion and exclusion criteria are described in detail in the study protocol both on patient- and site-level.

All analyses are performed on the intention-to-treat (ITT) analysis set that comprises patients and months from all randomized sites.

8 Handling of Missing Values, Missing Data, and Outliers

8.1 Missing Values, and Missing Data

Missing values are assumed to occur at random. Given the nature of the aggregated data, no imputation of missing entries is performed, and all analyses are based on complete case sets.

8.2 Outliers and implausible entries

Outliers and implausible entries are identified and, if required, corrected by the clinical project management. In addition to that, entries for the respective month are deleted if the count of patients with a specific event exceeds the count of patients, i.e., if for any month

- `Transm_Patient` > `Aufnahmen_Neo`
- `Transmission_2MRGN_Patient_SAE` > `Aufnahmen_Neo`
- `Anzahl_kl_Sepsis_allg` > `Aufnahmen_Neo`
- `Anzahl_BKpos_Sepsis_allg` > `Aufnahmen_Neo`
- `Anzahl_2MRGN_LOS` > `Aufnahmen_Neo`

In addition, values in the respective variables are deleted if

- the count of days with blocked beds in a month [`Tage_Bettensperrung`] exceeds 31 or
- the percentage of days with antibiotics treatment per patient days [`ATB_Tage_pro_Patiententag`] < 0.

9 Statistical Analyses/Methods

9.1 Blinded Data Review Meeting

Given the aggregated nature of the data, no blinded data review is performed.

9.2 Subject Disposition

There will be a clear accounting of all sites that participated in the study using tables and figures. The number of sites which were randomized, and that entered and completed each phase of the study will be provided as well as the reasons for all post-registration discontinuations. A flow chart according to the CONSORT statement will be prepared. The number of patients being admitted to the site and screened for multidrug-resistant organisms will be added.

A description of the cohort will be given using baseline characteristics.

9.3 Analysis of Primary Outcome Variables

Based on previous data, we assume a correlation within sites and phases of maximally 0.01. Given that the previously observed correlation is very low, the time lag between the two trial phases is long and different patients are included in the two trial phases at each site, no carry-over effects are assumed. We thus are not expecting that effects of one treatment phase would influence the second period after cross-over, so that data from two phases at one site are regarded as independent.

For the primary analysis, we estimate a GEE model, taking a possible correlation within the phases at one site (cluster as defined above) into account. Assuming a very low intra-cluster correlation, we use the independence structure for the working correlation. To obtain an estimate of the risk difference, an identity link is used. To test for non-inferiority, a margin of 0.05 has been defined.

Outcome variable	`Anzahl_gramNeg_nurLOS, Anzahl_gramNeg_nurLOS_negativ`
Endpoint	Gram-negative BSI
Effect measure	Risk difference
**Primary Estimand**	
Population	Newborns requiring intensive care in one of the participating NICUs undergoing KRINKO screening and being treated with hygiene measures
Treatment	Standard hygiene measures (hand disinfection plus gloves and gowns, CO) versus hand disinfection only (IV)
Hypothesis	Non-inferiority, i.e.<br>H_0: Risk for gram-negative BSI in IV – Risk for gram-negative BSI in CO group $\geq$ 0.05

	H_1: Risk for gram-negative BSI in intervention group – Risk for gram-negative BSI in control group < 0.05
Significance level	0.05 (one-sided)
Intercurrent Events	None
Strategy	n.a.
Adjustments for covariates	None
Main estimator	Risk difference is estimated from a GEE logistic regression model with identity link using the following command from the R-library geepack: `geeglm(cbind(Anzahl_gramNeg_nurLOS, Anzahl_gramNeg_nurLOS_negativ) ~ Randomisierung, family = binomial(link = "identity"), id = Cluster, corstr = "independence")` with the variable `Randomisierung` indicating the phase (CO vs. IV) From this model, the 95% confidence interval for the risk difference and the one-sided p-value are estimated.
Missing data	Analyses are based on complete cases
Interpretation for the primary estimand	If non-inferiority is statistically significant it is shown that newborns requiring intensive care being treated in a NICU with a hand disinfection only-protocol do not have a higher risk of gram-negative infections than corresponding newborns treated in a NICU with a standard protocol.
**Interim analysis**	n.a.
**Sensitivity analyses: logistic regression**	As sensitivity analysis, a logistic regression model is estimated assuming independence of the observations within one phase at one site using the following R-command: `glm(gramNeg_nurLOS ~ Randomisierung, family = binomial(link = "identity"))` with the variable `Randomisierung` indicating the phase (CO vs. IV) From this, the one-sided descriptive p-value and the 95% confidence interval are estimated.
Hypothesis	n.a.
Significance level	Descriptive analysis only
**Sensitivity analyses: Confounding**	As further sensitivity analyses, the same models are estimated as above with simultaneously taking possible confounders into account: `geeglm(cbind(Anzahl_gramNeg_nurLOS, Anzahl_gramNeg_nurLOS_negativ) ~ Randomisierung + Confounder, family = binomial(link = "identity"), id = Cluster, corstr = "independence")` with the variable `Randomisierung` indicating the phase (CO vs. IV) and Confounder being one of the three variables<br>• Percentage of shifts with understaffing in the month [`GBA_Prozent`]<br>• Count of days with blocked beds in the month [`Tage_Bettensperrung`]<br>• additional workload in the NICU by patients > 28 days of age [`Aufnahme_Päd`].<br>The risk difference with regard to the intervention is estimated accordingly with 95% confidence intervals.
Hypothesis	n.a.
Significance level	Descriptive analysis only
**Supplementary analysis**	n.a.
**Subgroup analysis**	n.a.

9.4 Analysis of Secondary Outcome Variables

9.4.1 Efficacy Outcome Variables on the level of months

For the following binary variables, the observational unit is the month:

- At least one suspected transmission of 2MRGN [Transm_2MRGN_j_n]
- At least one of any relevant transmissions [Transmission_j_n]
- At least one started reserve antibiotic [Beg_Reserve_ATB]

For these, the below analyses are performed:

Outcome variable	Transm_2MRGN_j_n, Transmission_j_n, Beg_Reserve_ATB
Endpoint	Per month, at least one suspected transmission of 2MRGN, at least one of any relevant transmissions, at least one started reserve antibiotic
Effect measure	Risk difference
**Secondary Estimand**	
Population	Months in NICUs with newborns requiring intensive care, undergoing KRINKO screening and being treated with hygiene measures
Treatment	Standard hygiene measures (hand disinfection plus gloves and gowns, CO) versus hand disinfection only (IV)
Hypothesis	n.a.
Significance level	Descriptive analysis only
Intercurrent Events	None
Strategy	n.a.
Adjustments for covariates	None
Main estimator	Risk differences are estimated from a GEE logistic regression model with identity link using the following command from the R-library geepack: `geeglm(Outcome ~ Randomisierung, family = binomial(link = "identity"), id = Cluster, corstr = "independence")` with the variable Randomisierung indicating the phase (CO vs. IV) and Outcome being one of the three variables<br>• At least one suspected transmission of 2MRGN [Transm_2MRGN_j_n]<br>• At least one of any relevant transmissions [Transmission_j_n]<br>• At least one started reserve antobiotic [Beg_Reserve_ATB]<br>The risk difference with regard to the intervention is estimated accordingly with 95% confidence intervals.
Missing data	Analyses are based on complete cases

In addition, the following continuous variables are assessed monthly:

- Number of started antibiotics cycles [Anzahl_beg_ATB]
- Percentage of days with antibiotic therapy [ATB_Tage_pro_Patiententag]

The analyses are performed analogously to the above analyses, but instead of logistic models, linear models are estimated with the log-transformed dependent variable as follows:

Outcome variable	log(Anzahl_beg_ATB), log(ATB_Tage_pro_Patiententag)

Endpoint	Per month, the number of started antibiotics cycles, per month, the percentage of days with antibiotic therapy
Effect measure	Mean difference on log-scale, i.e., percent change
**Secondary Estimand**	
Population	Months in NICUs with newborns requiring intensive care, undergoing KRINKO screening and being treated with hygiene measures
Treatment	Standard hygiene measures (hand disinfection plus gloves and gowns, CO) versus hand disinfection only (IV)
Hypothesis	n.a.
Significance level	Descriptive analysis only
Intercurrent Events	None
Strategy	n.a.
Adjustments for covariates	None
Main estimator	Risk differences are estimated from a GEE linear regression model using the following command from the R-library geepack:<br>`geeglm(Outcome ~ Randomisierung, family = gaussian, id = Cluster, corstr = "independence")`<br>with the variable `Randomisierung` indicating the phase (CO vs. IV) and Outcome being one of the two variables<br>• log-transformed number of started antibiotics cycles [`log(Anzahl_beg_ATB)`]<br>• log-transformed percentage of days with antibiotic therapy [`log(ATB_Tage_pro_Patiententag)`]<br>The percent change with regard to the intervention is estimated accordingly with 95% confidence intervals.
Missing data	Analyses are based on complete cases

9.4.2 Efficacy Outcome Variables on the level of patients

For the following binary variables, the count of patients without the event is reconstructed:

- Transmission of 2MRGN [`Anzahl_gramNeg_nurLOS_negativ`]
- Any transmission [`Transm_Patient_negativ`]
- Any BSI [`Anzahl_BKpos_Sepsis_allg_negativ`]
- 2MRGN BSI [`Transmission_2MRGN_Patient_SAE_negativ`]
- Clinical infection [`Anzahl_kl_Sepsis_allg_negativ`]

For these, the below analyses are performed:

Outcome variable	`Anzahl_gramNeg_nurLOS, Anzahl_gramNeg_nurLOS_negativ, Transm_Patient, Transm_Patient_negativ, Anzahl_BKpos_Sepsis_allg, Anzahl_BKpos_Sepsis_allg_negativ, Transmission_2MRGN_Patient_SAE, Transmission_2MRGN_Patient_SAE_negativ, Anzahl_kl_Sepsis_allg, Anzahl_kl_Sepsis_allg_negativ`
Endpoint	Transmission of 2MRGN, any transmission, any BSI, 2MGRN BSI, clinical infection
Effect measure	Risk difference

**Secondary Estimand**	
Population	Newborns requiring intensive care in one of the participating NICUs undergoing KRINKO screening and being treated with hygiene measures
Treatment	Standard hygiene measures (hand disinfection plus gloves and gowns, CO) versus hand disinfection only (IV)
Hypothesis	n.a.
Significance level	Descriptive analysis only
Intercurrent Events	None
Strategy	n.a.
Adjustments for covariates	None
Main estimator	Risk differences are estimated from a GEE logistic regression model with identity link using the following command from the R-library geepack: `geeglm(cbind(events, nonevents) ~ Randomisierung, family = binomial(link = "identity"), id = Cluster, corstr = "independence")`<br>with the variable `Randomisierung` indicating the phase (CO vs. IV) and `events` and `nonevents` being one of the five variable pairs<br>• `Anzahl_gramNeg_nurLOS, Anzahl_gramNeg_nurLOS_negativ`<br>• `Transm_Patient, Transm_Patient_negativ`<br>• `Anzahl_BKpos_Sepsis_allg, Anzahl_BKpos_Sepsis_allg_negativ`<br>• `Transmission_2MRGN_Patient_SAE, Transmission_2MRGN_Patient_SAE_negativ`<br>• `Anzahl_kl_Sepsis_allg, Anzahl_kl_Sepsis_allg_negativ`<br>The risk difference with regard to the intervention is estimated accordingly with 95% confidence intervals.
Missing data	Analyses are based on complete cases

9.4.3 Safety/Tolerability Outcome Variables

No further safety variables were assessed. The extent of exposure is indirectly assessed via the separate survey of the clinical project management. In this, the site-specific use of disinfection [Desinf_Verb] and the site-specific costs for gloves and gowns [Kosten] are recorded. These will be summarized per phase.

10 Software

For analyses, SAS version 9.4 and R Version 4.1.3 or higher will be used.
