# Supplementary material for: Extended Barrier Precautions vs Hand Hygiene Alone and Neonatal Sepsis in Intensive Care Patients: The BALTIC Cluster-Randomized Clinical Trial
Source: JAMA Netw Open. 2026 May 15;9(5):e2612759. doi: 10.1001/jamanetworkopen.2026.12759 (PMC13179548; doi:10.1001/jamanetworkopen.2026.12759)
Supplement: Supplement 2. — eFigure 1. Study Design of the BALTIC Trial eFigure 2. Center Specific End Points During Study Period eFigure 3. Center Specific Colonization And Transmissions [file jamanetwopen-e2612759-s002.pdf]

Supplemental Online Content

Faust K, Strecker F, Haug C, et al. Extended barrier precautions vs hand hygiene alone and neonatal sepsis in intensive care patients: the BALTIC cluster-randomized clinical trial. *JAMA Netw Open*. 2026;9(5):e2612759. doi:10.1001/jamanetworkopen.2026.12759

eFigure 1. Study Design of the BALTIC Trial

eFigure 2. Center Specific End Points During Study Period

eFigure 3. Center Specific Colonization And Transmissions

This supplemental material has been provided by the authors to give readers additional information about their work.

© 2026 Faust K et al. *JAMA Network Open*.

Supplemental Figure 1

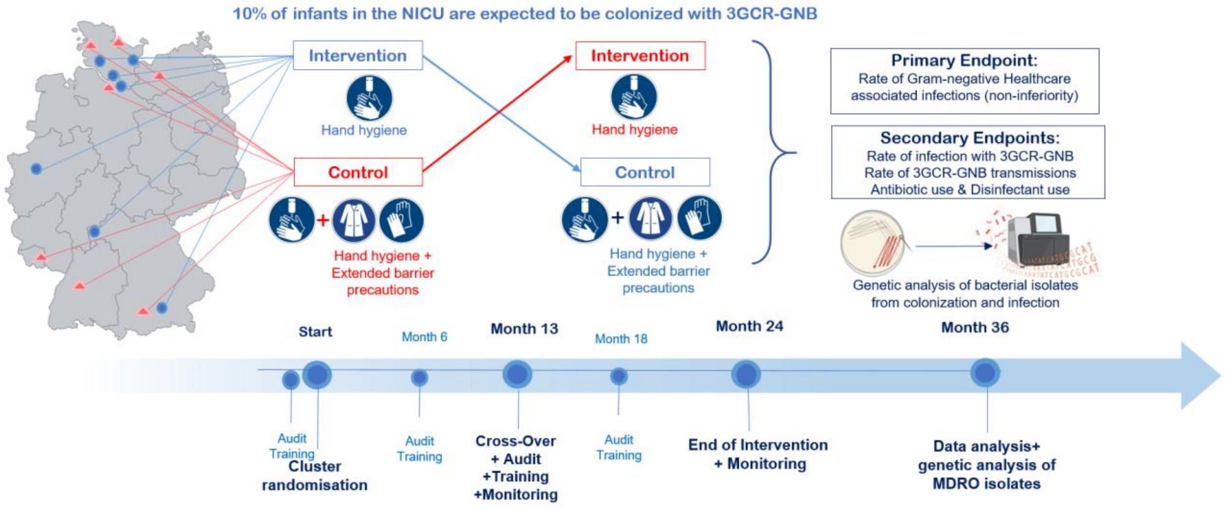

Legend Supplemental figure 1: Study design of the BALTIC trial MDRO multidrug-resistant organism, 3GCR-GNB, 3rd generation cephalosporin resistant gram negative bacteria, © C. Härtel

© 2026 Faust K et al. *JAMA Network Open*.

Supplemental Figure 2

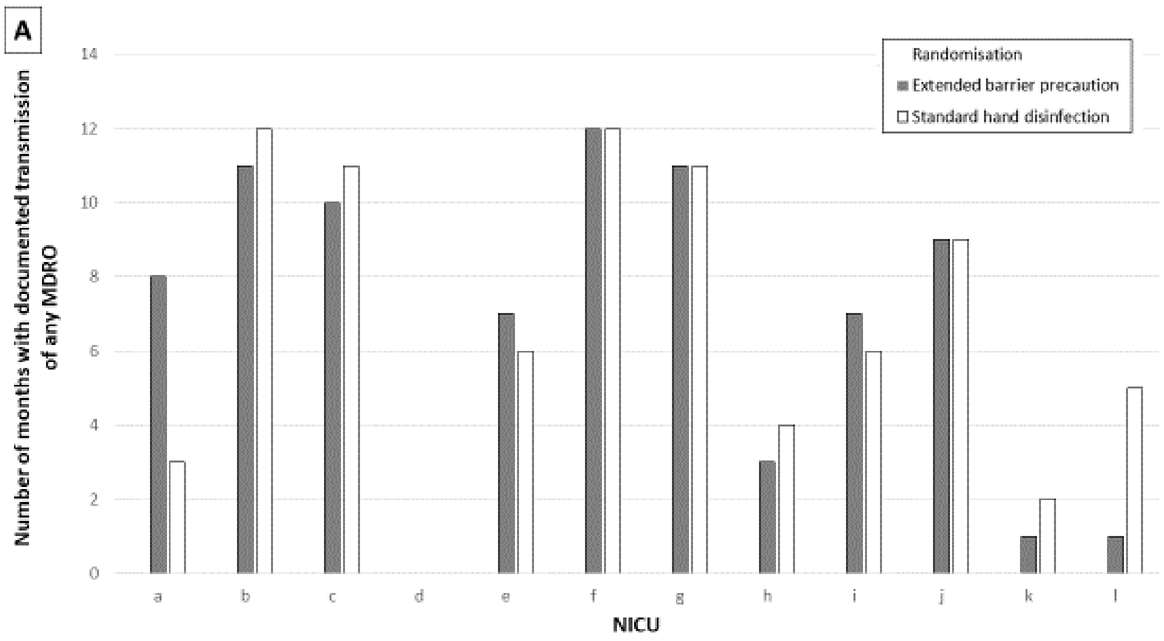

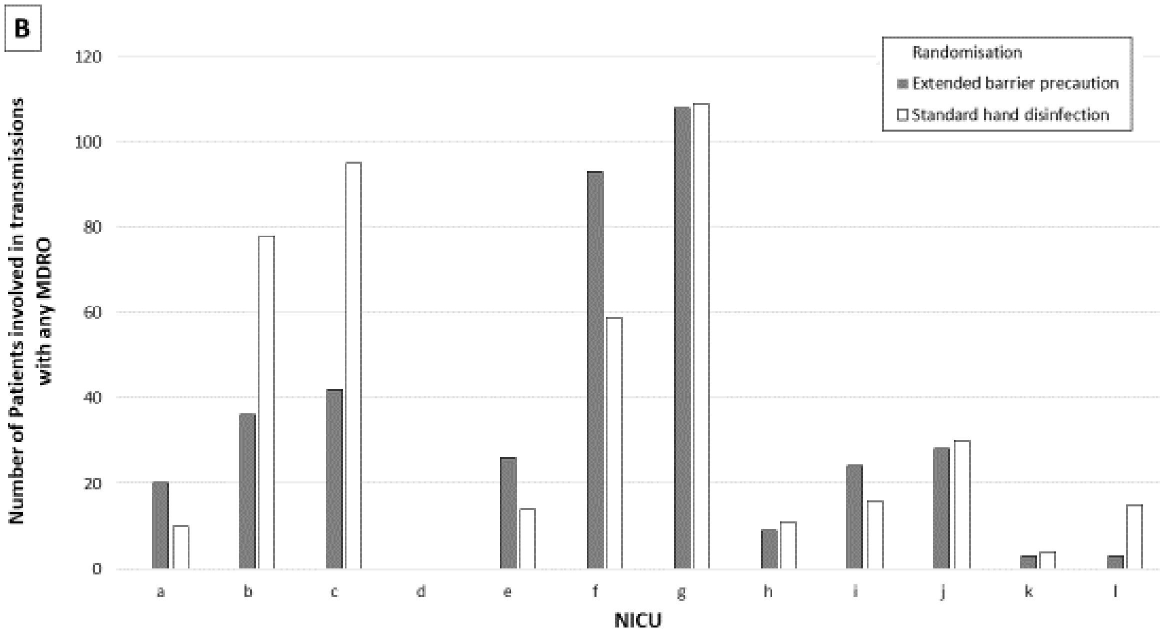

© 2026 Faust K et al. *JAMA Network Open*.

C

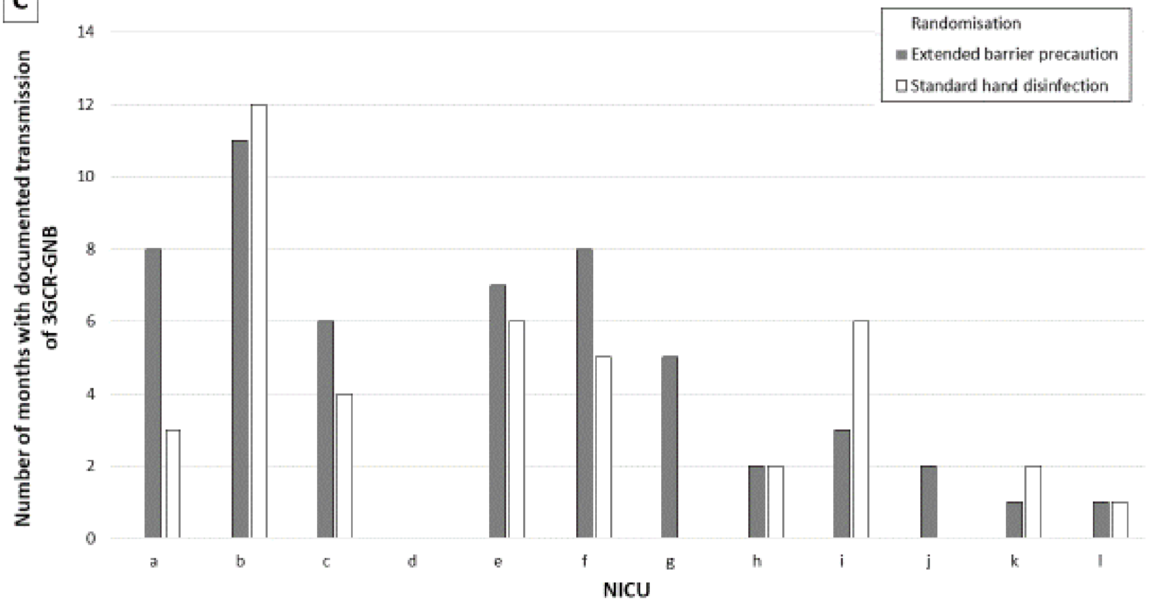

D

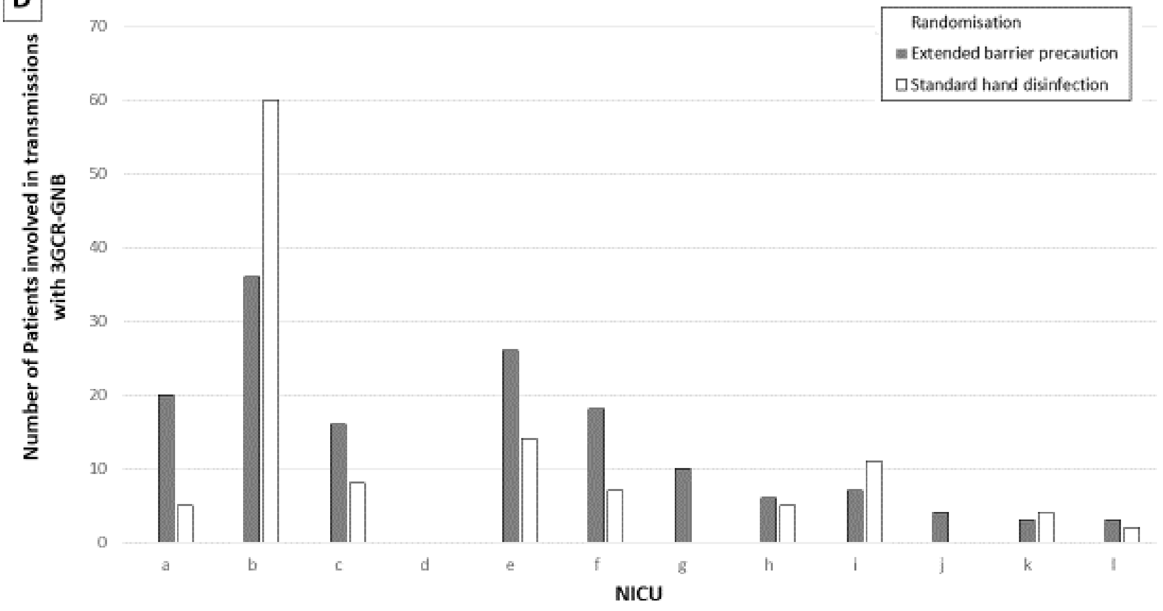

© 2026 Faust K et al. *JAMA Network Open*.

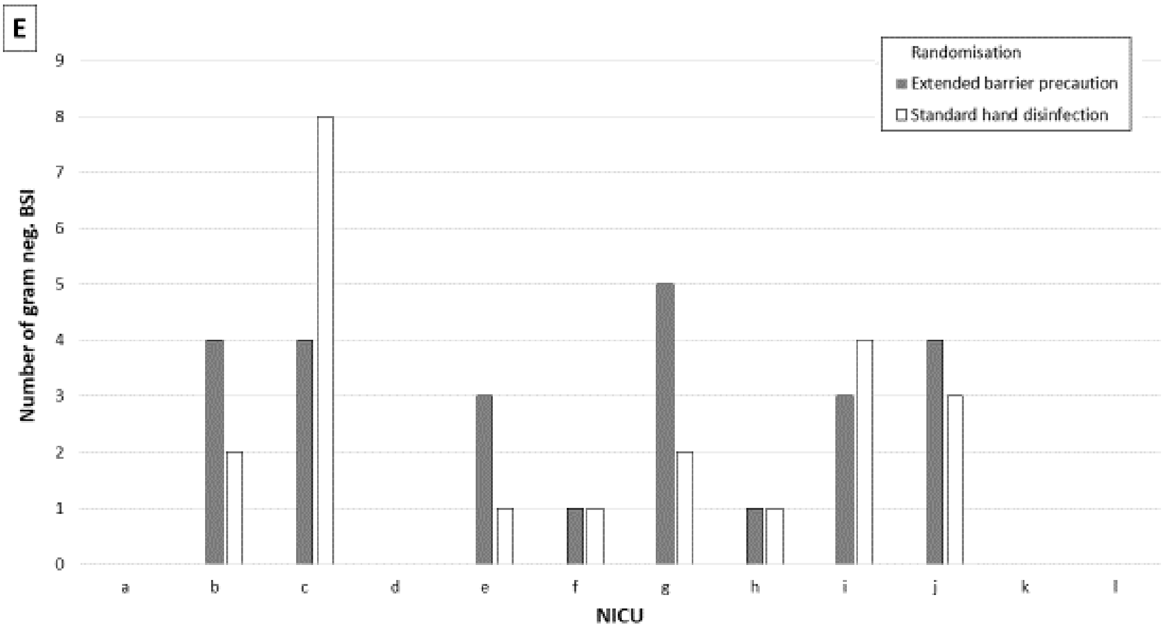

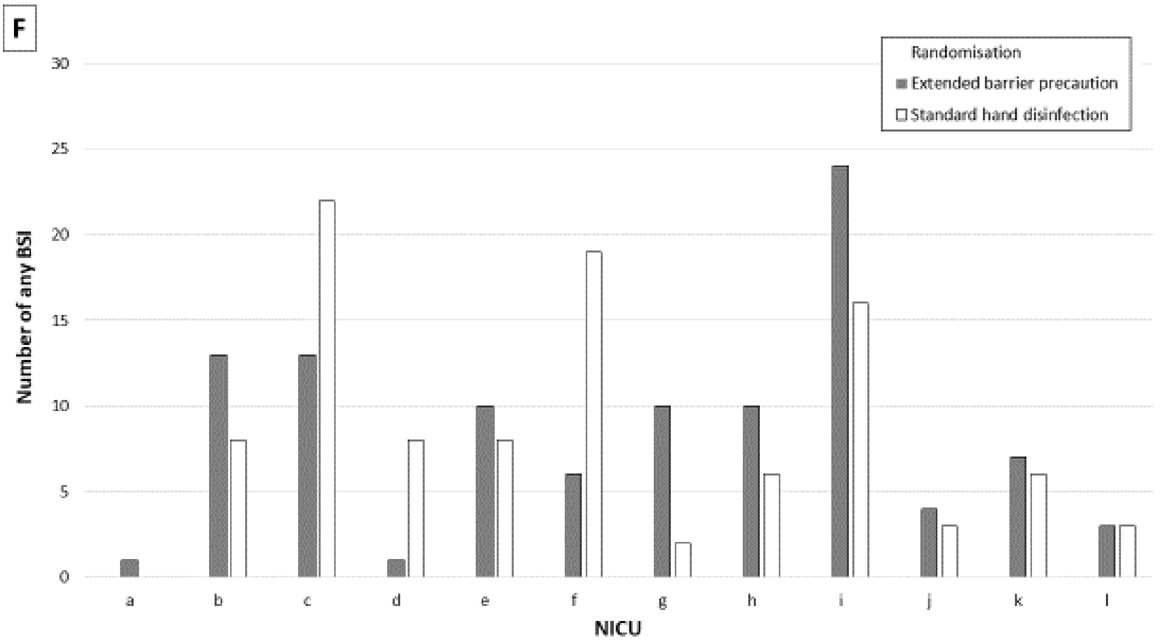

© 2026 Faust K et al. *JAMA Network Open*.

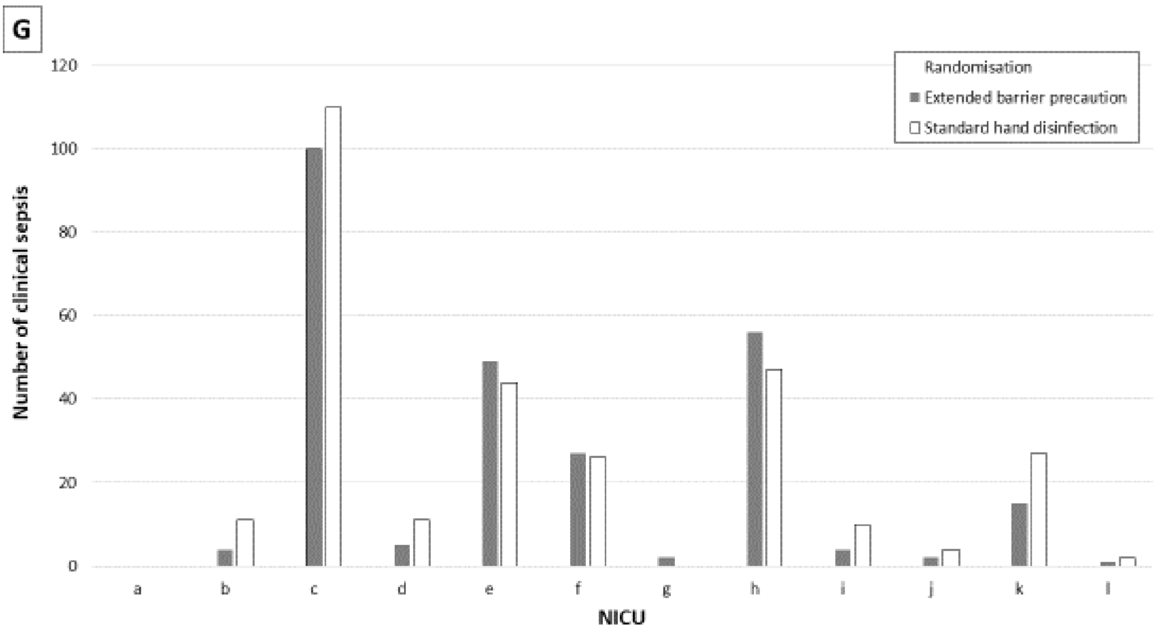

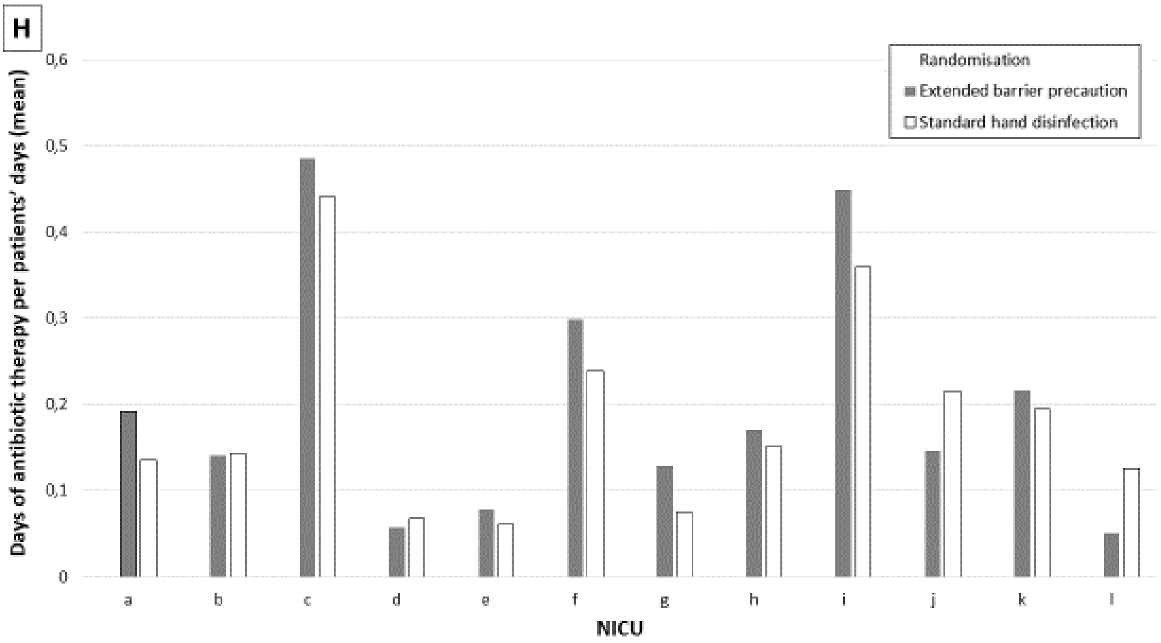

Legend Supplemental Figure 2: Center specific endpoints during study period depending on the NICU and randomization

- A+B: Number of months with recorded transmissions during study period (24months)
  - A: Transmissions of any MDRO:
  - B: Transmissions of 3GCR-GNB
- C+D: Total number of patients involved in a transmission depending on the NICU
  - C: Transmissions of any MDRO:
  - D: Transmissions of 3GCR-GNB
- E: Number of GN-BSI
- F+G: Number of documented cases of sepsis
  - F: Number of any BSI
  - G: Number of clinical sepsis
- H: Use of antibiotics: Days with antibiotic therapy in relation to patients' days (mean)

© 2026 Faust K et al. *JAMA Network Open*.

Supplemental Figure 3

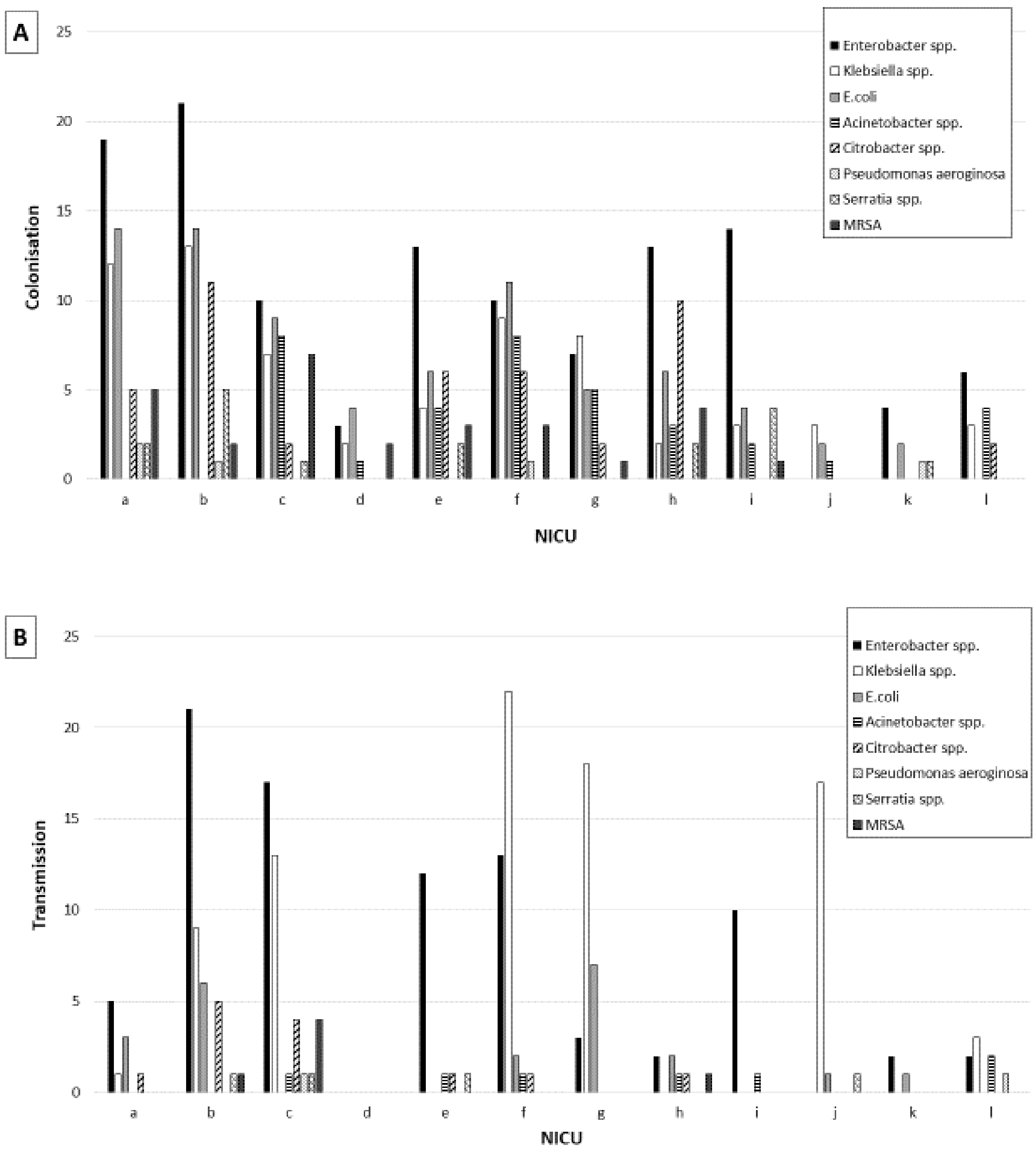

Legend Supplemental Figure 3: Center specific colonization and transmissions depending on the NICU

A: Colonization with MDRO depending on the NICU: Number of months with documented colonization of a specific pathogen (any MDRO) depending on the NICU

B: Transmission of MDRO depending on the NICU: Number of months with documented Transmission of a specific pathogen (any MDRO) depending on the NICU

© 2026 Faust K et al. *JAMA Network Open*.
