# Supplementary material for: Extended Barrier Precautions vs Hand Hygiene Alone and Neonatal Sepsis in Intensive Care Patients: The BALTIC Cluster-Randomized Clinical Trial
Source: JAMA Netw Open. 2026 May 15;9(5):e2612759. doi: 10.1001/jamanetworkopen.2026.12759 (PMC13179548; doi:10.1001/jamanetworkopen.2026.12759)
Supplement: Supplement 3. — Data Sharing Statement [file jamanetwopen-e2612759-s003.pdf]

Data Sharing Statement

Faust. Extended Barrier Precautions vs Hand Hygiene Alone and Neonatal Sepsis in Intensive Care Patients. *JAMA Netw Open*. Published May 15, 2026.
doi:10.1001/jamanetworkopen.2026.12759

Data
Additional Information: DRKS00019103 German Clinical Trial Registry
Data available: Yes
Data types: Data (not involving human participants)
How to access data: Request to corresponding author
When available: With publication

Supporting Documents
Document types: None

Additional Information
Who can access the data: researchers whose proposed use of the data has been approved
Types of analyses: any purpose
Mechanisms of data availability: with investigator support
Any additional restrictions: none
